# Antero-posterior ectoderm patterning by canonical Wnt signaling during ascidian development

**DOI:** 10.1371/journal.pgen.1008054

**Published:** 2019-03-29

**Authors:** Stacy Feinberg, Agnès Roure, Julie Piron, Sébastien Darras

**Affiliations:** Sorbonne Université, CNRS, Biologie Intégrative des Organismes Marins (BIOM), Banyuls/Mer, France; California Institute of Technology, UNITED STATES

## Abstract

Wnt/β-catenin signaling is an ancient pathway in metazoans and controls various developmental processes, in particular the establishment and patterning of the embryonic primary axis. In vertebrates, a graded Wnt activity from posterior to anterior endows cells with positional information in the central nervous system. Recent studies in hemichordates support a conserved role for Wnt/β-catenin in ectoderm antero-posterior patterning at the base of the deuterostomes. Ascidians are marine invertebrates and the closest relatives of vertebrates. By combining gain- and loss-of-function approaches, we have determined the role of Wnt/β-catenin in patterning the three ectoderm derivatives of the ascidian *Ciona intestinalis*, central nervous system, peripheral nervous system and epidermis. Activating Wnt/β-catenin signaling from gastrulation led to a dramatic transformation of the ectoderm with a loss of anterior identities and a reciprocal anterior extension of posterior identities, consistent with studies in other metazoans. Surprisingly, inhibiting Wnt signaling did not produce a reciprocal anteriorization of the embryo with a loss of more posterior identities like in vertebrates and hemichordate. Epidermis patterning was overall unchanged. Only the identity of two discrete regions of the central nervous system, the anteriormost and the posteriormost regions, were under the control of Wnt. Finally, the caudal peripheral nervous system, while being initially Wnt dependent, formed normally. Our results show that the *Ciona* embryonic ectoderm responds to Wnt activation in a manner that is compatible with the proposed function for this pathway at the base of the deuterostomes. However, possibly because of its fast and divergent mode of development that includes extensive use of maternal determinants, the overall antero-posterior patterning of the *Ciona* ectoderm is Wnt independent, and Wnt/β-catenin signaling controls the formation of some sub-domains. Our results thus indicate that there has likely been a drift in the developmental systems controlling ectoderm patterning in the lineage leading to ascidians.

## Author summary

The Wnt/β-catenin pathway is a system of cell-cell communication. It has an ancient origin in animals and plays multiple roles during embryogenesis and adult life. In particular, it is involved in determining, in the vertebrate embryo, the identity of the different parts of the body and their relative positions along the antero-posterior axis. We have investigated in an ascidian (or sea squirt) species, a marine invertebrate that is closely related to vertebrates, whether this pathway had a similar role. Like in vertebrates, activating Wnt/β-catenin led to a posteriorization of the embryo with a loss of anterior structures. By contrast, unlike vertebrates, ascidian embryos formed rather normally following Wnt/β-catenin inactivation. Since hemichordates (or acorn worms), earlier divergent invertebrates, use Wnt/β-catenin in a manner comparable to vertebrates, it is in the ascidian lineage that changes have occurred. Consequently, ascidians build an antero-posterior axis, very similarly organized to that of vertebrates, but in a different way.

## Introduction

Ascidians belong to the tunicates, the sister group of the vertebrates. Together with the cephalochordate (amphioxus) and vertebrate phyla they form the super-phylum of chordates whose specific body plan includes a notochord and a dorsal hollow neural tube during embryonic life. Comparative developmental studies between these three phyla is essential for elaborating evolutionary scenarios explaining the emergence of chordates and their diversification [1–4]. Ascidians are particularly puzzling organisms since they took a significantly different evolutionary path from other chordates resulting in divergent morphological, embryological and genomic features. Their development is fast and stereotyped with very few cells; and ascidian genomes have undergone compaction and extensive rearrangements when compared to vertebrates and amphioxus. This raises the question of whether developmental mechanisms controlling typical chordate structure formation are conserved between ascidians and other chordates. In particular, ascidian embryos are the emblematic examples for the concept of mosaic development. However, it is well known that cell-cell communication is involved in cell fate determination, yet possibly at only short distances (*i*.*e*. neighboring cells) [5–7].

Wnt signaling, one of the pathways present in animals, allows cells to communicate through the secretion of the Wnt ligands that bind to their cognate Frizzled receptors. It is involved in a wide range of biological processes during embryogenesis and adult homeostasis [8,9]. The canonical Wnt pathway (cWnt) that uses the protein β-catenin as a central mediator to control target gene transcription is extensively involved in axis formation during the development of many metazoans [10,11]. Three discrete developmental processes contribute to antero-posterior (AP) axis formation in bilaterians: germ layer specification, AP patterning and posterior growth. During cleavage/blastula stages, nuclear accumulation of β-catenin in the vegetal hemisphere specifies endomesoderm in several phyla (nemerteans, echinoderms, hemichordates and ascidians) [12–15]. A similar function for the specification of the endoderm at the oral pole of cnidarians suggests that this constitutes an ancient function at the base of metazoans [16,17]. In vertebrates, a posterior to anterior gradient of activity provides cells with positional information and patterns the central nervous system (CNS) [18–21]. A recent study in hemichordates demonstrated that this function for cWnt is conserved at the base of the deuterostomes [22]. Finally, in both insects and vertebrates, Wnt signaling controls body elongation during posterior growth [23].

Posterior growth does not exist in ascidians since the embryo elongation at the improperly named tailbud stages occurs through cell division and rearrangement without proper addition of new tissue from a growth zone [24]. Embryonic axes are determined very early and can be identified in the fertilized egg before first cleavage [5]. cWnt participates in endomesoderm formation along the animal-vegetal axis [13,25]. The AP axis is orthogonal and determined following ooplasmic movements that localize asymmetric cleavage determinants to the posterior. A consequence for AP patterning is that anterior (so called a-line) and posterior (b-line) ectoderm precursors have intrinsically different potentials in response to neural induction as soon as they arise at the 8-cell stage [26]. Interestingly, not only the CNS but also the epidermis is patterned along the AP axis; and this patterning also involves signals from vegetal tissues [27]. The transcription factor FoxA-a is the anterior determinant that establishes the early a-line *versus* b-line potentials [28,29]. Since direct transcriptional FoxA-a targets are Wnt antagonists–a-line expressed *Sfrp1/5* and *Ror* genes–there is a potential role for Wnt signaling in ectoderm AP patterning. Moreover, the AP identity of the two sensory pigment cells within the CNS is controlled by Wnt signaling [30]. However, a global function for Wnt in ectoderm AP patterning has not been investigated; and this is the topic of the present manuscript.

Sequencing and annotation of the ascidian *Ciona robusta* (formerly known as *C*. *intestinalis* type A) has revealed a complement for Wnt signaling compatible with a functional pathway [31–34]. A recent phylogenetic analysis has shown that the ten Wnt ligands found in the *Ciona* genome correspond to 10 out of the 13 families present at the base of chordates, with the loss of *Wnt1*, *Wnt4* and *Wnt8* [35]. Their spatio-temporal expression has been described throughout embryogenesis for eight of them, but only a few show a restricted pattern (*Wnt3*, *Wnt5*, *Wnt6* and *Wnt7*) [35–39]. In particular, they do not display a staggered expression in the posterior of the embryo as observed for many metazoans including vertebrates and amphioxus (reviewed in [10], [35]). The only possible similarity would be the expression of the four above mentioned ligands in caudal muscle at cleavage/gastrula stages and the epidermal expression of *Wnt5* at the posterior tip of the forming tail. At the opposite pole of the embryo, the Wnt antagonists, *Sfrp1/5* and *Ror* genes, are expressed in the anterior ectoderm as described above [28,29,36,37]. The *C*. *robusta* genome contains five Frizzled receptors [33]. The expression pattern is known for three of them (*Frizzled1/2/7* and *Frizzled5/8* are maternally and ubiquitously expressed; *Frizzled4* is expressed in the ectoderm from the 16-cell stage and later in various discrete regions), but does not allow us to predict where and when Wnt signaling is active [36].

In the present study, we have combined ectopic activation and down-regulation of the cWnt pathway to assess the effects on AP patterning of the *C*. *intestinalis* embryonic ectoderm. Activating cWnt from gastrulation leads to a loss of anterior ectoderm that is converted in posterior ectoderm. By contrast, inhibiting cWnt has varying effects depending on the ectoderm derivatives. Epidermis AP patterning is unchanged. The CNS is largely unaffected, except for its anterior and posterior ends, suggesting a function of cWnt in refining a global AP pattern that is defined by other means. Finally, the early definition of the caudal peripheral nervous system (PNS) requires cWnt signaling but redundant mechanisms allow proper differentiation of this tissue. Consequently, while the *Ciona* ectoderm displays a sensitivity to cWnt activation that is compatible with the expected function for cWnt at the base of deuterostomes, cWnt is only marginally required for ectoderm AP patterning.

## Results

### Timing of cWnt activity on ectoderm patterning

**Fig 1:**
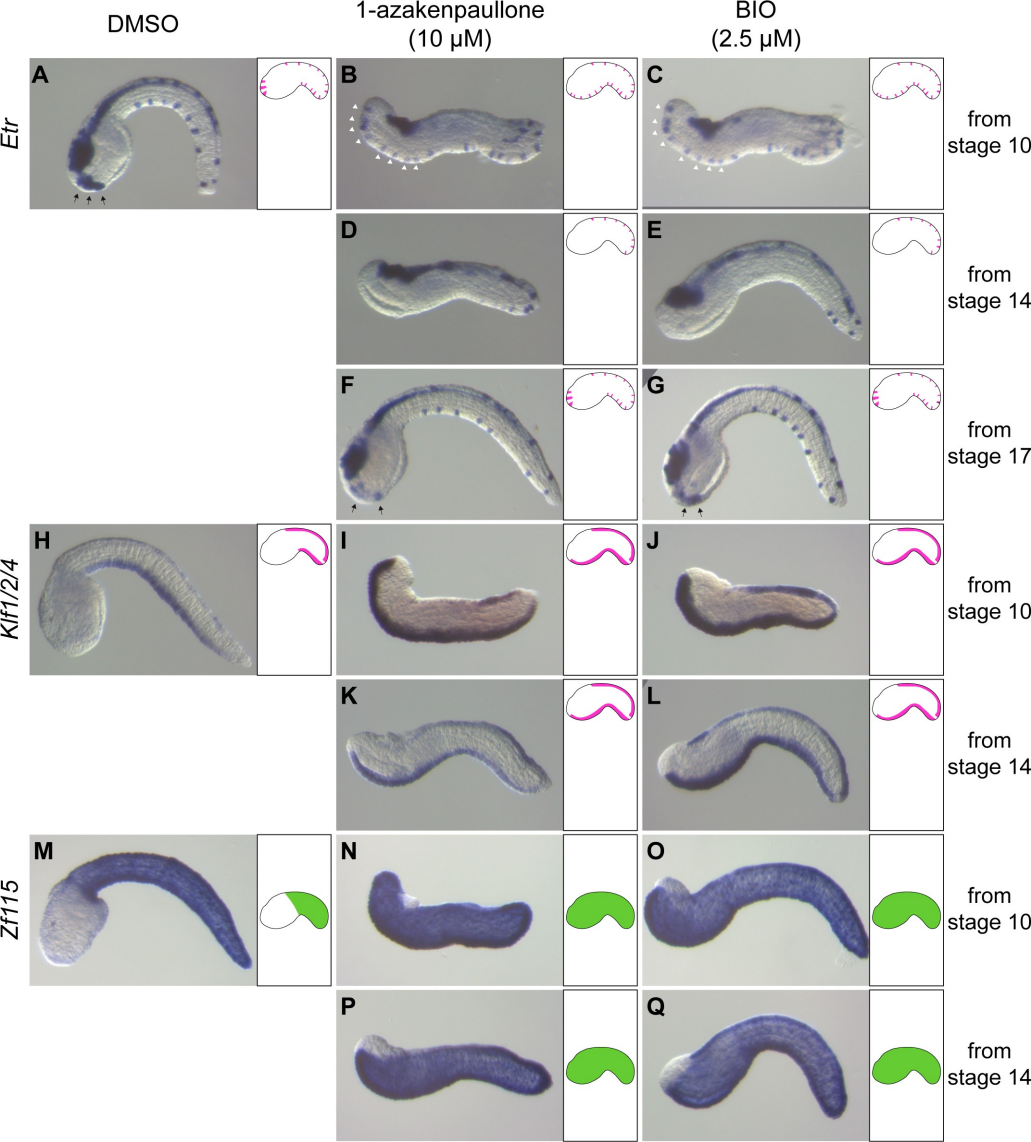
Consequences of activating cWnt at different developmental stages on PNS and epidermis formation. Embryos were treated with 10 μM 1-azakenpaullone or 2.5 μM BIO from initial gastrula (stage 10; B, C, I, J, N and O), from early neurula (stage 14; D, E, K, L, P and Q) or from initial tailbud stages (stage 17; F and G) until fixation at late tailbud stages (stages 23/24). Expression of *Etr* (A-G), *Klf1/2/4* (H-L) and *Zf115* (M-Q) was determined by *in situ* hybridization. Black arrows point to palp neurons, white arrowheads indicate ectopic *Etr* staining in the ventral trunk epidermis midline. Embryos are oriented with dorsal to the top and anterior to the left. On the right side of each picture, a schematic embryo depicts our interpretation of the expression pattern according to each tissue: PNS (pink at the top) and epidermis (green in the middle). Number of experiments: one for B, C, F, G, K and L; two for A, D, E, H, I, J and M to Q.

LiCl or small molecule inhibitors of Gsk3β have been previously used in ascidian embryos to activate the cWnt pathway [13,25,40]. We have further developed such treatments using two distinct inhibitors, 1-azakenpaullone and BIO [41,42]. These inhibitors were tested at two doses (5 and 10 μM for 1-azakenpaullone; 1 and 2.5 μM for BIO). The results presented here correspond to the highest dose for each molecule, conditions leading to fully penetrant and identical phenotypes for both inhibitors. As expected, early treatments led to ectopic endoderm formation as revealed by staining for endoderm specific endogenous alkaline phosphatase activity (S1 Fig). Treatments starting at the 32-cell stage or later produced embryos with a dramatically abnormal morphology but without ectopic endoderm, allowing us to determine effects on ectoderm patterning without interfering with germ layer formation. A previous report has suggested that activating the cWnt pathway interferes with epidermal sensory neuron (ESN) formation along the AP axis in the tail [40]. We reproduced the reported results: a loss of anterior ventral ESN formation (revealed by the expression of *Etr* at late tailbud stages) when the treatment was initiated at early neurula stages (stage 14) and an absence of effect when the treatment was initiated at initial tailbud stages (stage 17) (Fig 1D–1G). However, when the treatment was initiated at the onset of gastrulation (stage 10), we observed ectopic ESNs located in the ventral trunk midline (Fig 1B and 1C). Caudal ESNs are known to arise from a neurogenic territory characterized by the expression of *Klf1/2/4* [43]. The presence of ectopic ESNs in the trunk upon cWnt activation was accompanied by the ectopic expression of *Klf1/2/4* in the ventral trunk midline (Fig 1I–1L), suggesting that these ectopic ESNs arise from an ectopic neurogenic territory. Interestingly, *Klf1/2/4* ectopic expression was also observed in treatments starting at stage 14 while *Etr* expression was repressed. To further investigate the apparent posteriorization of the ectoderm following cWnt activation, we determined the expression of *Zf115*, a gene with a marked restricted expression in the tail epidermis [44]. *Zf115* was ectopically expressed in the entire trunk epidermis for both of our early treatments (Fig 1N–1Q) but not for the latest treatment (S2 Fig). To further delineate the sensitivity of the ectoderm to cWnt activation, we performed 30 min treatments at various developmental stages and assessed the expression of both *Etr* and *Zf115*. Ectopic expression of *Etr* in the ventral trunk was observed when such short treatments were performed during gastrulation (stages 10 to 13), but not later (S2 Fig). The loss of anterior ventral tail *Etr* expression described above was not observed in the pulse treatments suggesting a longer exposure time may be required. Ectopic *Zf115* expression in the trunk was observed for all pulse treatments with a reducing effect as the treatment was delayed: treatment at the onset of gastrulation led to an ectopic expression in the entire trunk while later treatments led to an extension limited to the posterior trunk (S2 Fig).

Above results suggest that cWnt activation converts trunk ectoderm into tail ectoderm with a maximum sensitivity during early gastrulation.

### cWnt activation using small molecule inhibitors posteriorizes the ectoderm

**Fig 2:**
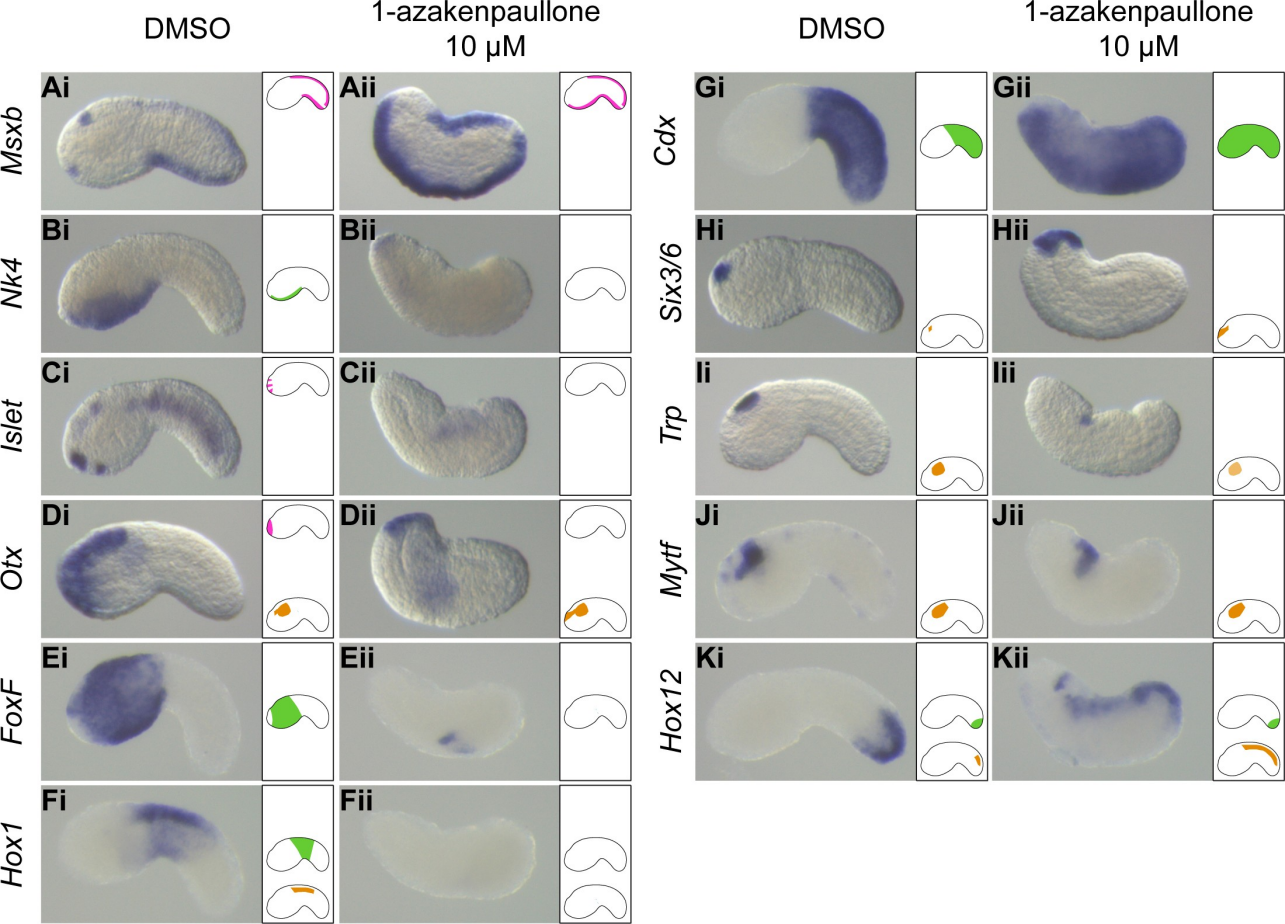
Activating cWnt using small molecule inhibitors posteriorizes the ectoderm. Embryos were treated with 10 μM 1-azakenpaullone from stage 10 (initial gastrula), fixed at stage 19/20 (early tailbud) and processed for *in situ* hybridization to determine the expression pattern of *Msxb* (A), *Nk4* (B), *Islet* (C), *Otx* (D), *FoxF* (E), *Hox1* (F), *Cdx* (G), *Six3/6* (H), *Trp* (I), *Mytf* (J) and *Hox12* (K). DMSO-treated control embryos (i) and 1-azakenpaullone-treated embryos (ii). Embryos are oriented with dorsal to the top and anterior to the left. On the right side of each picture, a schematic embryo depicts our interpretation of the expression pattern according to each tissue: PNS (pink at the top), epidermis (green in the middle) and CNS (orange at the bottom). Number of experiments: one for *Islet*, *FoxF*, *Hox1*, *Cdx*, *Trp*, *Mytf* and *Hox12*; two for *Msxb*, *Nk4*, *Otx* and *Six3/6*.

In this section, we will determine what are the effects of activating cWnt from gastrulation on all three ectoderm derivatives: the epidermis, the peripheral and the central nervous system. We have thus examined by *in situ* hybridization the expression of a panel of AP markers for the ectoderm at early tailbud stages (stage 19/21) following 1-azakenpaullone or BIO treatment from initial gastrula (stage 10). Both drugs led to similar effects (Figs 2 and S3). Interestingly, while we observed a dose response on the morphology of embryos treated with 1-azakenpaullone, the effect on the AP markers examined remained consistent for all doses (S4 Fig). We did not observe a graded effect similar to what was observed when the treatment was staggered over timed intervals (S2 Fig).

#### Peripheral nervous system

As described previously, ectopic ESN formation in the ventral midline of the trunk ectoderm following cWnt activation is accompanied by the formation of an ectopic neurogenic territory. This result is confirmed by the ectopic expression at early tailbud stages of the transcription factor coding gene *Msxb* that is necessary and sufficient for neurogenic territory and ESN formation [45] (Figs 2A and S3). Ectopic ventral "tail midline" in the trunk led to a loss of trunk ventral ectoderm identity as revealed by the loss of expression of several markers such as *Nk4*, *Nkx-A*, *Bmp2/4* and *Smad6/7* (Figs 2B and S3). A similar situation was not observed on the dorsal side, the neurogenic markers, *Msxb* and *Klf1/2/4*, and the neuronal marker *Etr* did not extend anteriorly (Figs 1B–1L and 2A and S3).

At the very anterior end of the embryo, peripheral sensory neurons of the palps express the genes *Etr* and *Islet* (Figs 1A and 2C). Expression of both genes was lost following cWnt activation (Figs 1B, 1C and 2C and S3). Accordingly, anterior epidermal expression of *Ror-a* and *Otx* that delineates the palps territory was also abolished (Figs 2D and S3).

#### Epidermis

The loss of anterior identity was not limited to the anteriormost ectoderm that is epidermal and neurogenic (palp neurons). *FoxF*, that marks the entire trunk epidermis except the anteriormost part, was also lost following cWnt activation (Fig 2E). Epidermal expression of *Hox1* at the trunk/tail junction and in the anterior tail was also suppressed (Fig 2F). In contrast, expression of the two tail epidermis markers, *Zf115* and *Cdx*, was extended to the entire trunk epidermis (Figs 1M–1Q and 2G and S3). The epidermal expression of the very posterior gene *Hox12* was weakly extended anteriorly (Fig 2K). However, such weak ectopic expression did not demonstrate that the epidermis acquired posteriormost identity. In summary, we conclude that trunk epidermis acquired a tail epidermis identity when cWnt was activated.

#### Central nervous system

We have observed different gene behaviors in response to cWnt activation according to the AP level where they are normally expressed. For example, *Six1/2* and *Six3/6* expression at the anteriormost edge of the CNS (sensory vesicle) has expanded anteriorly (Figs 2H and S3 and S4). Expression of other genes in the sensory vesicle, *Etr*, *Ror-a*, *Otx*, *Trp*, *Gbe1* and *Mytf*, was maintained albeit possibly at weaker levels (Figs 1A–1C, 2D and 2I–2J and S3). The tail nerve cord was still present as revealed by the expression of *KH*.*C7*.*391* (S3 Fig), but its patterning was modified. While *Hox1*, normally expressed in the anterior nerve cord, was not expressed, *Hox12*, a marker for the posterior tip of the nerve cord, was ectopically expressed anteriorly (Fig 2F and 2K).

#### Non-ectodermal derivatives

We have seen that the endoderm formed somewhat normally following cWnt activation after cleavage stages (S1 Fig), however, its patterning was affected. *Cdx*, which is normally expressed in the tail endoderm (the endodermal strand), was ectopically expressed in the ventral trunk endoderm; while ventral endodermal trunk expression of *Nk4*, *Nkx-A* and *Bmp2/4* was lost (S3 Fig). Mesodermal derivatives appeared to form normally since expression of *FoxF* in cardiopharyngeal progenitors (the trunk ventral cells), *Ferritin* in the tail muscle, and *Brachyury* and *Tgf-β* in the notochord were maintained (Figs 2E and S3 and S4). Notochord AP patterning was likely modified. Endogenous alkaline phosphatase activity is found in the trunk endoderm and posteriormost notochord in swimming larvae (S4 Fig). This activity was observed throughout the notochord following 1-azakenpaullone treatment. Similarly, *Tgf-β*, whose normal expression increases anteriorly to posteriorly in the notochord, was found to have a uniform pattern following cWnt activation (S4 Fig). Thus, the formation of both endodermal and mesodermal derivatives appears to be largely unaffected by cWnt activation, although they are likely posteriorized.

### cWnt activation using overexpression posteriorizes the ectoderm

**Fig 3:**
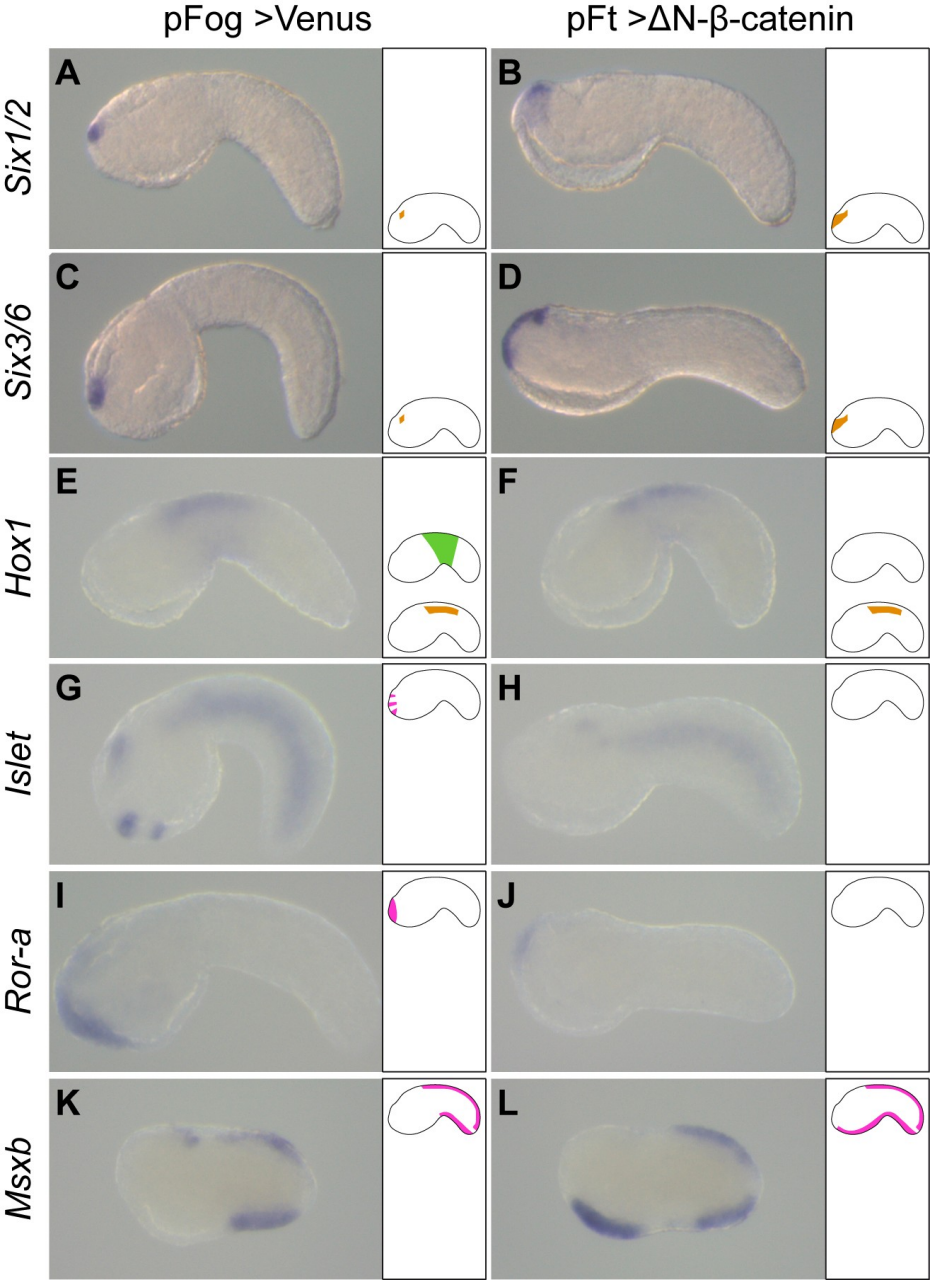
Activating cWnt by overexpression of ΔN-β-catenin phenocopies Gsk3 inhibitor treatment. Embryos were electroporated and fixed for *in situ* hybridization at initial tailbud stages (stage 18, K and L) and early tailbud stages (stage 21, A to J). Control pFog>Venus-electroporated embryos (A, C, E, G, I and K) and pFt>ΔN-β-catenin-electroporated embryos (B, D, F, H, J and L). Following ΔN-β-catenin overexpression, note the anterior extension of *Six1/2* (B) and *Six3/6* (D), the repression of epidermal expression of *Hox1* (F), the repression of *Islet* (H) and *Ror-a* (J) in the palp forming region, and the ectopic expression of *Msxb* (L) in the ventral trunk epidermis. Embryos are oriented with dorsal to the top and anterior to the left. On the right side of each picture, a schematic embryo depicts our interpretation of the expression pattern according to each tissue: PNS (pink at the top), epidermis (green in the middle) and CNS (orange at the bottom). Number of experiments: one for *Hox1*, *Islet* and *Ror-a*; two for *Six1/2*, *Six3/6* and *Msxb*.

To verify the specificity towards Wnt/β-catenin signaling of the above results, we overexpressed Wnt5, a ligand normally restricted to the posterior ectoderm [36], throughout the ectoderm using DNA electroporation. This led to ectopic expression of the tail midline markers *Msxb*, *Klf1/2/4* and *Nkx-C* in the ventral trunk epidermis (S5 Fig). However, the embryo morphology was severely affected, making gene expression analysis tedious. We turned to overexpression of ΔN-β-catenin, a version of β-catenin that is deleted from the N-terminal domain (containing Gsk3β phosphorylation sites) and that behaves as a dominant active form [40]. We could reproduce the results obtained using Gsk3 inhibitor treatments: ectopic expression of *Six1/2*, *Six3/6*, *Msxb* and *Klf1/2/4* (Figs 3B, 3D and 3L and S5), and repression of the epidermal expression of *Hox1*, *Islet* and *Ror-a* (Fig 3F, 3H and 3J). The CNS expression of *Hox1* was not affected since we targeted the ectoderm using the promoter of the *Fucosyl transferase* gene [40]; CNS *Hox1* positive cells originate from vegetal lineages and do not express this gene. These observations strengthen our findings that AP patterning defects result from direct action of Wnt/β-catenin signaling.

### Overlap between cWnt and Bmp signals defines the tail ventral midline

**Fig 4:**
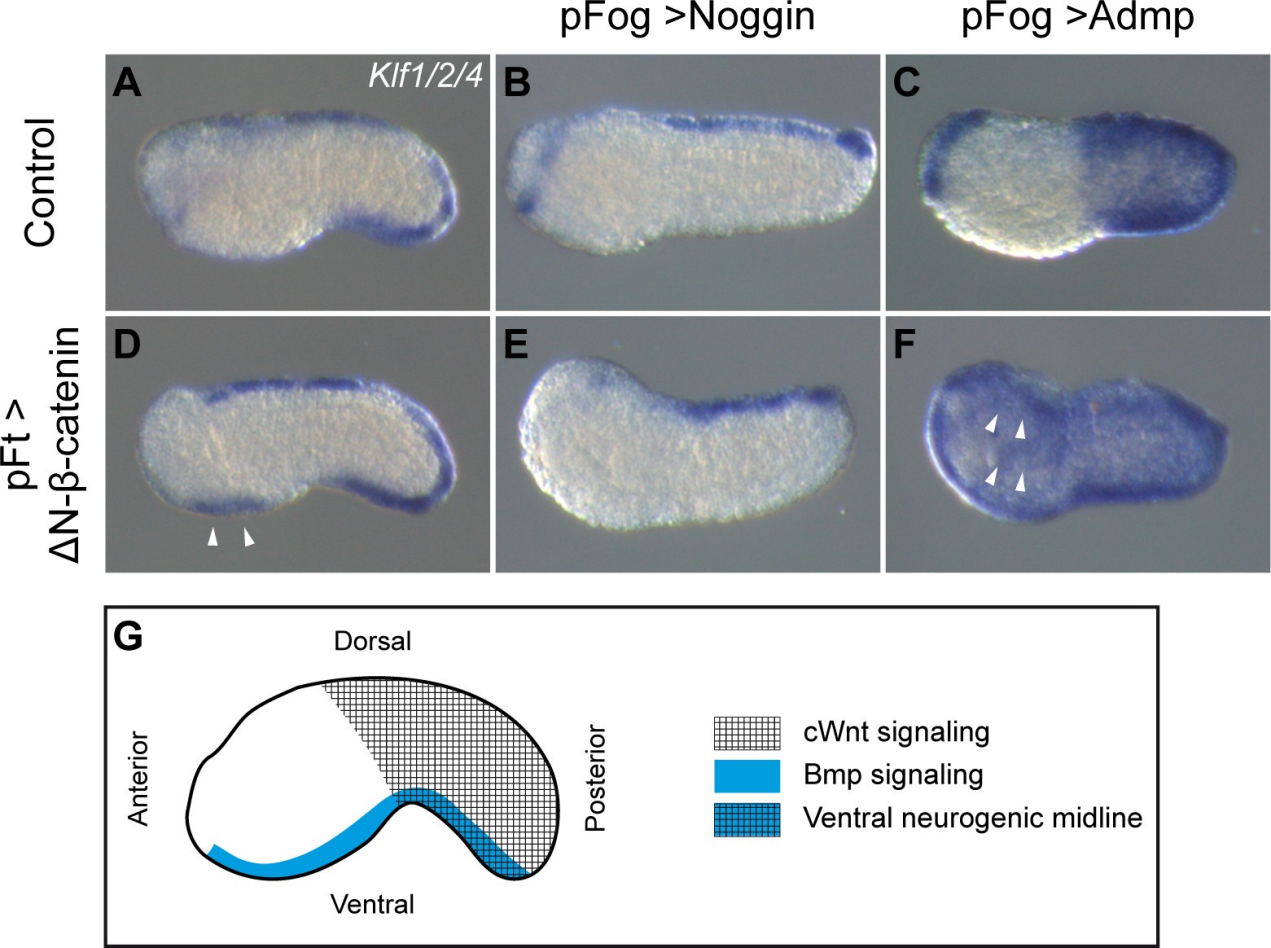
A combination of Bmp and Wnt signals defines the tail ventral neurogenic midline. (A) Control unelectroporated embryo. Embryos were electroporated with pFog>Noggin (B), pFog>Admp (C), pFt>ΔN-β-catenin (D), pFog>Noggin + pFt>ΔN-β-catenin (E), and pFog>Admp + pFt>ΔN-β-catenin (F), and fixed for *in situ* hybridization at initial tailbud stages (stage 18) for *Klf1/2/4*. Ectopic staining is highlighted by the white arrowheads. (G) Schematic proposing that the tail ventral neurogenic midline territory forms where both Wnt and Bmp pathways are active. One experiment was performed.

In previous results, we observed that activating cWnt led to the formation of an ectopic neurogenic territory in the ventral trunk epidermis. It is known that Bmp signaling is required to specify the tail ventral midline and that Bmp signaling is active throughout the ventral epidermis, both in the trunk and the tail [43,46]. We thus expressed Noggin, a secreted Bmp antagonist, together with ΔN-β-catenin. As predicted, ectopic expression of *Klf1/2/4* and *Msxb* was suppressed (Figs 4E and S6). As previously reported, when Bmp signaling was activated, *Klf1/2/4* and *Msxb* were ectopically expressed throughout the tail epidermis (Figs 4C and S6) [43,46]. Activation of cWnt signaling in addition to Bmp led to ectopic activation of both genes in the trunk epidermis as well (Figs 4F and S6), suggesting that combining cWnt and Bmp signals is sufficient to launch the tail ventral neurogenic program (Fig 4G).

### cWnt activity is dispensable for epidermis patterning

We have used the overexpression of two different proteins to block Wnt signaling. TcfΔC is a dominant negative form of the transcription factor Tcf that normally regulates transcription, together with β-catenin, downstream of the binding of a Wnt ligand to a Frizzled receptor. It contains a C-terminal deletion that eliminates the DNA binding domain of Tcf and has been previously used in *Ciona* to inhibit β-catenin nuclear activity during endomesoderm formation [25,47]. Sfrp1/5 is a naturally secreted antagonist of Wnt signaling that acts by sequestering Wnt ligands and thus preventing them from binding to Frizzled receptors [48]. Both molecules were overexpressed in the entire ectoderm using the promoters of the *Friend of gata* (*Fog*) or *Fucosyl transferase* (*Ft*) genes [40,49]. The following combinations produced the strongest phenotypes and were used in subsequent experiments: pFog>TcfΔC and pFt>Sfrp1/5.

**Fig 5:**
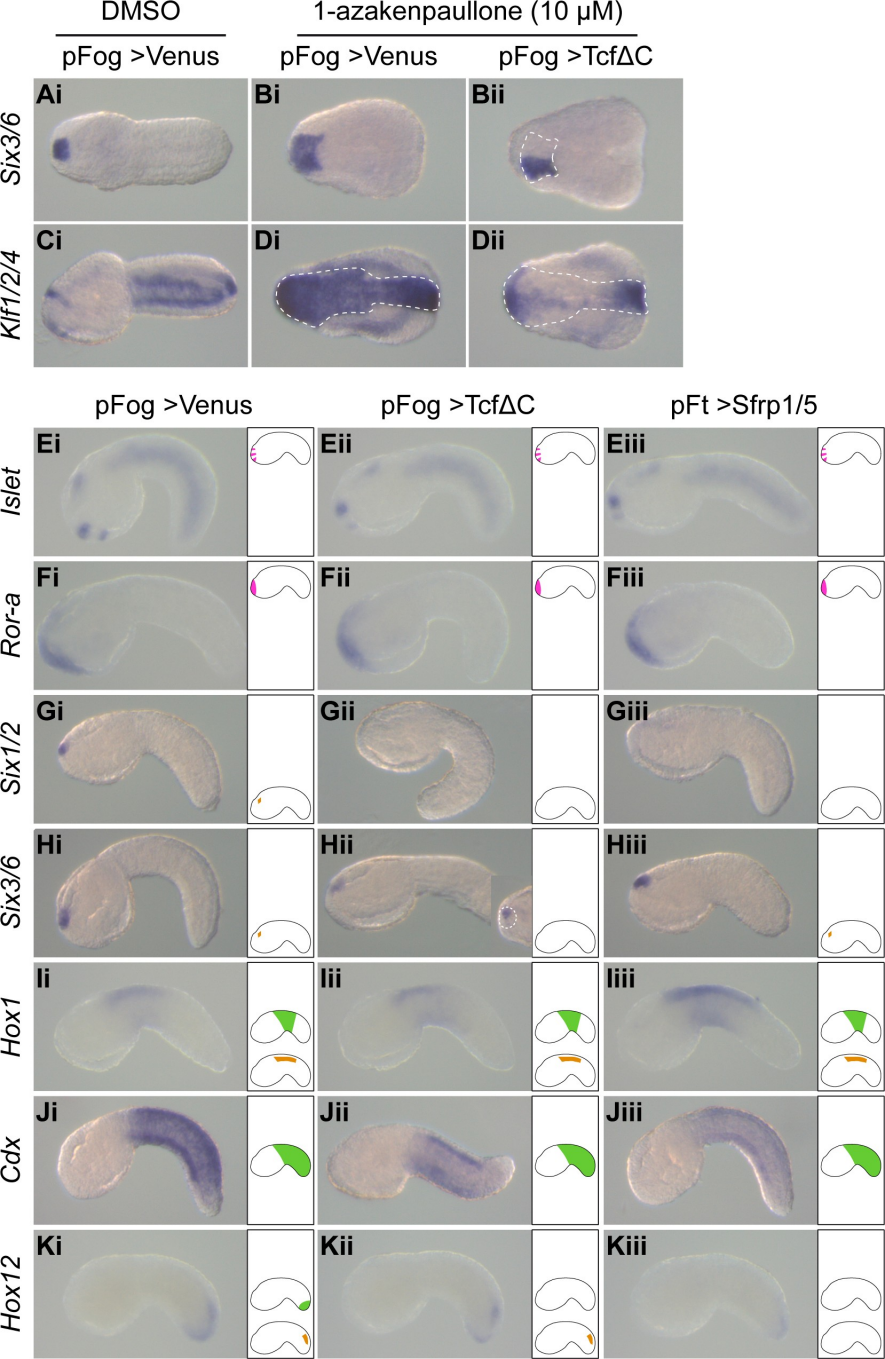
Effect of Wnt signaling inhibition on ectoderm AP markers. Embryos were electroporated with pFog>Venus (i), pFog>TcfΔC (ii), or pFt>Sfrp1/5 (iii), treated with DMSO (A, C) or 10 μM 1-azakenpaullone from stage 10 (initial gastrula) (B, D), and fixed for *in situ* hybridization at early tailbud stages (stage 21) for *Six3/6* (A, B and H), *Klf1/2/4* (C and D), *Islet* (E), *Ror-a* (F), *Six1/2* (G), *Hox1* (I), *Cdx* (J) and *Hox12* (K). Inset in Hii shows the trunk region of the same embryo in dorsal view (anterior to the left), with a remaining unilateral patch of expression resulting from the mosaic inheritance of the pFog>TcfΔC plasmid. Embryos are in dorsal view (A, B and Hii inset), ventral view (C and D) or lateral view oriented with dorsal to the top (E-K, except Hii inset) and anterior to the left. On the right side of pictures E to K, a schematic embryo depicts our interpretation of the expression pattern according to each tissue: PNS (pink at the top), epidermis (green in the middle) and CNS (orange at the bottom). Number of experiments: one for A to F; two for G and I; three or more for H, J and K.

We first determined the efficiency of our constructs by testing their ability to counteract the effect of Gsk3 inhibitor treatment. Overexpression of TcfΔC was sufficient to suppress the ectopic activation of both *Six3/6* and *Klf1/2/4* triggered by 1-azakenpaullone treatment (Fig 5A–5D). We could not perform the same assay for Sfrp1/5 since it acts upstream of Gsk3 inhibitors in the cWnt pathway. However, its overexpression had similar effects as TcfΔC overexpression did.

Given the robust phenotypes on epidermal expression following cWnt activation, we expected a strong reciprocal effect for Wnt inhibition: loss of posterior markers and posterior extension of anterior marker expression. We were surprised to see that epidermal expression of *Islet*, *Ror-a*, *Hox1* and *Cdx* was unchanged (Fig 5E, 5F, 5I and 5J), with possibly a weak reduction in levels of expression in the most affected embryos as depicted for *Cdx* on Fig 5J. The only clear difference we could detect was a repression of the epidermal expression of *Hox12* using both constructs (Fig 5Kii and 5Kiii), but we did not detect a concomitant posterior extension of *Cdx* into the tail tip (Fig 5J). Consequently, with the exception of *Hox12* and possibly the tail tip, epidermis AP patterning is largely unchanged following Wnt signaling inhibition.

### A limited portion of the CNS requires cWnt activity

We have determined, at tailbud stages, the expression of CNS genes whose expression was modified following cWnt activation: *Six1/2*, *Six3/6*, *Hox1* and *Hox12*. Both *Six1/2* and *Six3/6* were robustly repressed by pFog>TcfΔC, but only slightly downregulated by pFt>Sfrp1/5 in a minority of embryos (Fig 5G and 5H). This suggests that both genes could be positively regulated by cWnt signaling although in a ligand independent manner. Anterior tail nerve cord *Hox1* expression was unchanged (Fig 5I). Expression of *Hox12* in the posterior of the tail nerve cord was downregulated by pFt>Sfrp1/5 but unaffected by pFog>TcfΔC (Fig 5K). This difference possibly stems from the embryonic origin of the *Hox12* expressing cells that may be of vegetal origin (A-line). Consequently, they do not express the promoters used and as such, do not express the transgenes. Since Sfrp1/5 is a secreted molecule it can prevent these cells from receiving Wnt signals.

In summary, CNS patterning is regulated by Wnt signaling at only the anteriormost and the posteriormost regions of the axis.

### cWnt regulates medio-lateral anterior neural plate border patterning

**Fig 6:**
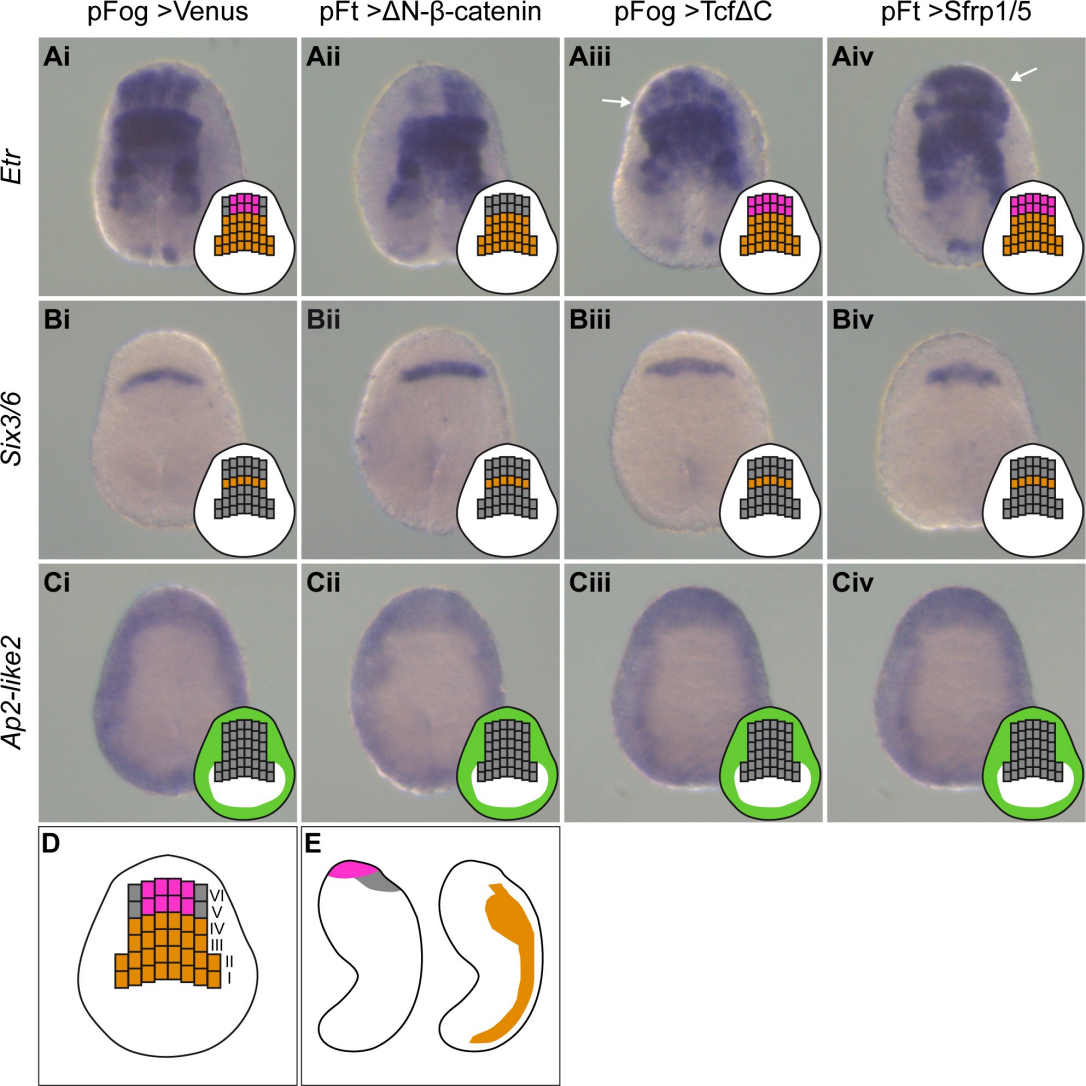
Regulation of anterior neural plate border patterning by Wnt signaling. (A-C) Embryos were electroporated with pFog>Venus (i), pFt>ΔN-β-catenin (ii), pFog>TcfΔC (iii), or pFt>Sfrp1/5 (iv), and fixed for *in situ* hybridization at early neurula stages (stages 13/14) for *Etr* (A), *Six3/6* (B) and *Ap2-like2* (C). Ectopic expression in the lateral anterior neural plate border is indicated by the white arrows. For each embryo, a schematic in the bottom right corner depicts the neural plate (rows I to IV) and the anterior neural plate border (rows V and VI) in grey, and gene expression according to tissue: PNS (pink), epidermis (green) and CNS (orange). The eight median cells expressing *Etr* in rows V and VI are the palp region precursors. Gene expression change is unilateral in Aii, Aiii and Aiv because of the mosaic inheritance of the electroporated plasmid. Embryos are in dorsal (neural plate) view with anterior to the top. (D-E) Schematic representation of the progeny of neural plate blastomeres (D) at tailbud stages (E): palp precursors in pink, aATENS precursors in grey and CNS precursors in orange (adapted from [52,69]). Dorsal view with anterior to the top in D, and lateral view with anterior to the top and ventral to the left in E. Number of experiments: one for *Six3/6*; two for *Etr* and *Ap2-like2*.

The above results prompted us to test the effects of Wnt signaling modulations on the early formation of the CNS at the neural plate stage (stages 13/14) (Fig 6). *Etr*, whose expression is found in the CNS precursors (rows I to IV according to [50]) and in the palp forming region at the medial anterior neural plate border (rows V and VI; Fig 6Ai and 6D), displayed a loss of expression in this latter territory following cWnt activation (Fig 6Aii). This corroborates previous results obtained at later stages with the markers *Ror-a*, *Otx* and *Islet*. However, the loss of this marker does not correspond to a conversion into more posterior neural tissue since the expression of *Six3/6*, which is immediately posterior to the anterior neural plate border, was unchanged (row IV; Fig 6B). Moreover, *Ap2-like2* expression was also unchanged and did not extend posteriorly (Fig 6C) suggesting that a conversion into epidermis had not occurred. When Wnt signaling was inhibited, *Etr* was ectopically expressed laterally in the anterior neural plate border (Fig 6Aiii and 6Aiv). These observations suggest that Wnt signaling regulates medio-lateral patterning of the anterior neural plate border. Importantly, as development proceeds, the medial part of the anterior neural plate border stained by *Etr* will form the very anterior palps region while the lateral part, *Etr* negative, will form the region immediately posterior containing anterior apical trunk ESNs (aATENs) (Fig 6D and 6E) [51,52].

### Posterior PNS formation is only initially dependent on cWnt

**Fig 7:**
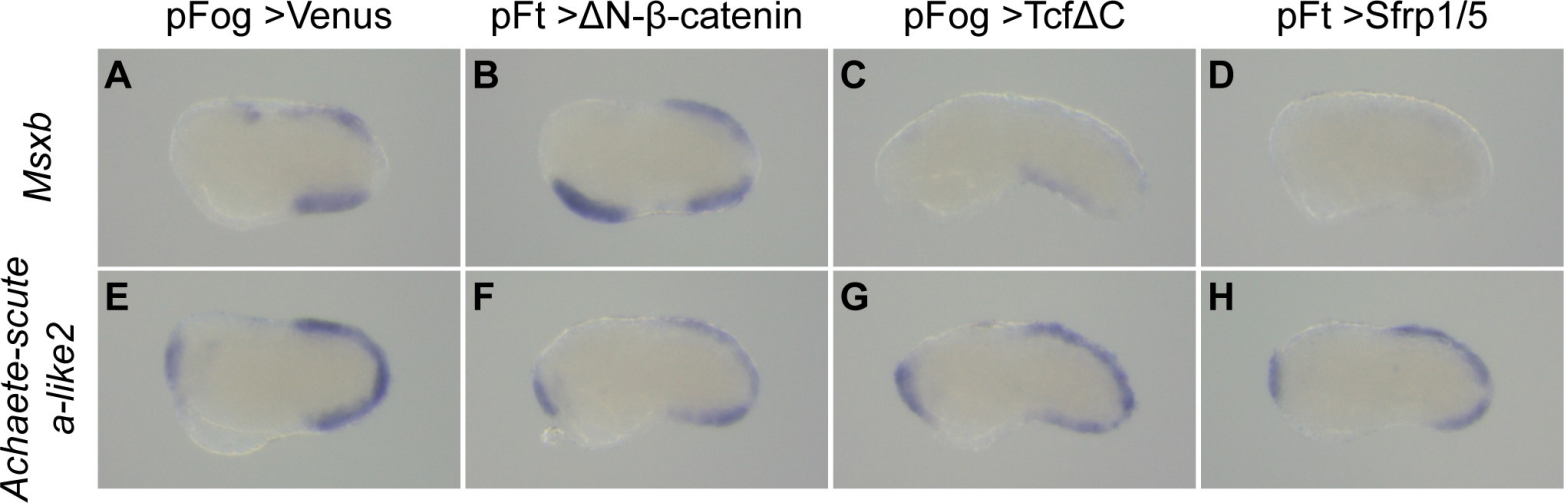
Differential regulation of tail PNS genes by Wnt signaling. Embryos were electroporated with pFog>Venus (A, E), pFt>ΔN-β-catenin (B, F), pFog>TcfΔC (C, G), and pFt>Sfrp1/5 (D, H), and fixed for *in situ* hybridization at initial tailbud stages (stage 18) for *Msxb* (A-D) and *Achaete-scute a-like2* (E-H). *Msxb* is positively regulated by cWnt signaling: ectopic expression in the ventral trunk epidermis upon activation (B) and downregulation in dorsal and ventral tail neurogenic regions upon inhibition (C, D). By contrast, *Achaete-scute a-like2* expression is unchanged for both cWnt activation or inhibition (F-H). Embryos are oriented with dorsal to the top and anterior to the left. Number of experiments: one for *Achaete-scute a-like2* and three or more for *Msxb*.

We next tested the requirement of Wnt signaling for tail PNS formation. In a reciprocal manner to their activation following cWnt activation (S5 Fig), the genes *Msxb*, *Klf1/2/4* and *Nkx-C* were strongly downregulated following either TcfΔC or Sfrp1/5 overexpression (Figs 7C, 7D and S7). Of these, *Msxb* was the most affected gene and displayed a complete loss of expression by *in situ* hybridization for the strongest phenotypes (Figs 7C, 7D and S8). We next assessed ESN formation using *Etr* as a marker. To avoid confusion with CNS staining, we only scored ventral ESNs. The number and location of ESNs is stochastically determined and so varies from embryo to embryo [43]. For the control embryos electroporated with pFog>Venus, we counted 6.8 ESNs on average (n = 44 embryos). The numbers for the experimental embryos were as follows: 6.8 ESNs for pFt>Sfrp1/5 (n = 46) and 5.8 for pFog>TcfΔC (n = 42). This suggests that Wnt signaling is not essential for tail PNS formation. A possible explanation comes from the observation of *Achaete-scute a-like2*, a transcription factor expressed in the tail neurogenic midlines [45]. Contrary to the other three genes examined, *Achaete-scute a-like2* expression was unchanged following either activation or inhibition of Wnt signaling (Fig 7E–7H). *Achaete-scute a-like2* could thus compensate the downregulation of other transcription factors and allow tail PNS formation when Wnt signaling is blocked.

## Discussion

**Fig 8:**
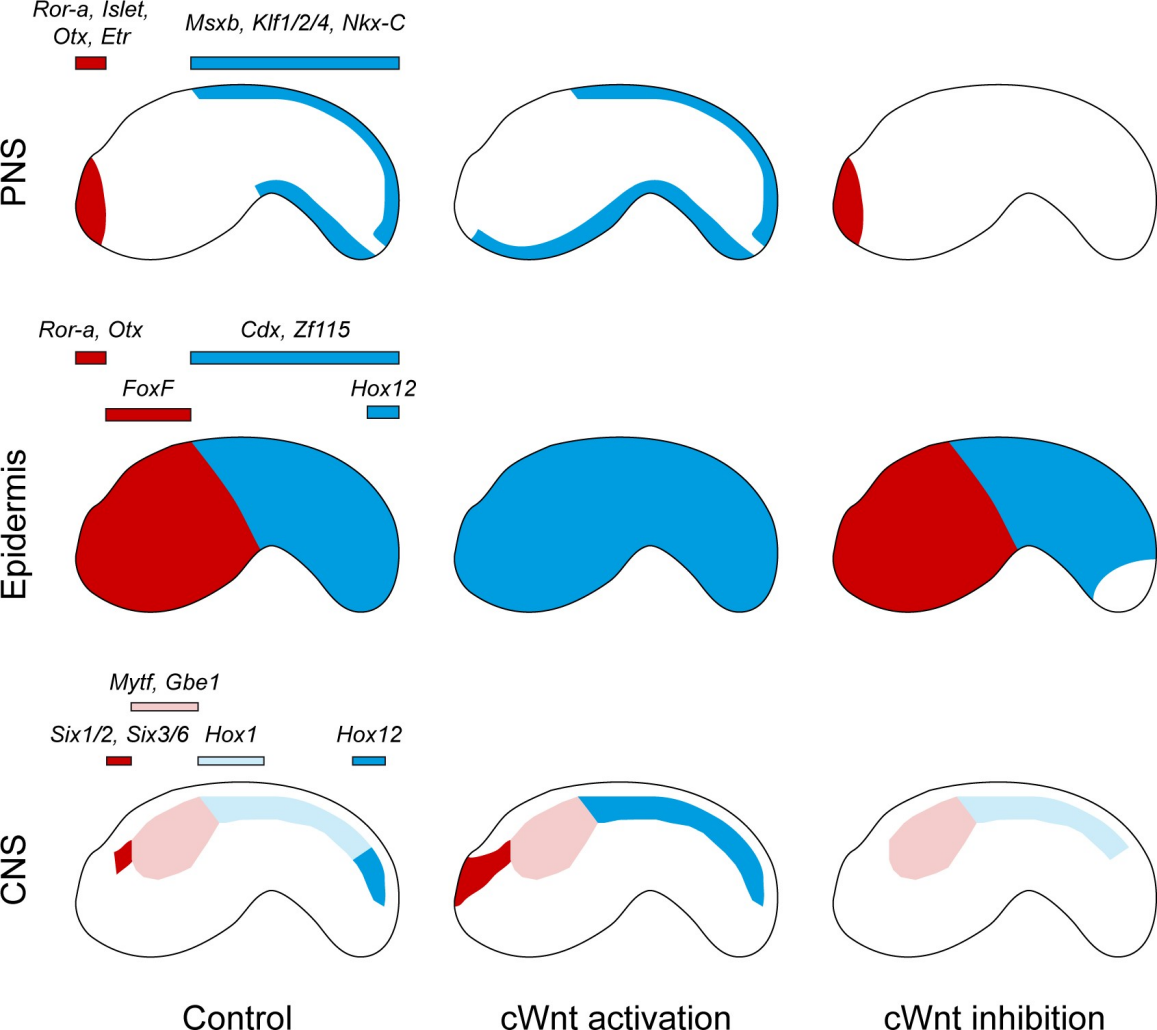
Summary of phenotypic effects of cWnt modulation on ectoderm AP patterning in *Ciona intestinalis*. Schematic representation of the results obtained in this study for the AP pattern of the PNS, the epidermis and the CNS following cWnt activation or inhibition. Red: anterior. Blue: posterior. Some of the key patterning genes whose expression has been determined in functional studies are listed above each domain.

The impacts of modulating cWnt activity on the AP pattern of the ectoderm are summarized in Fig 8. Activating cWnt using either pharmacological Gsk3 inhibitors or overexpression of a constitutively active β-catenin led to very dramatic modifications of the ectoderm AP axis. By analyzing the expression of markers that delineate broad AP domains of the epidermis and the neurogenic epidermis of the PNS (the anterior palp forming region and the caudal midlines), we observed a loss of anterior identity (*Otx*, *Ror-a*, *Islet* and *Etr* in the palp region; *FoxF* in the trunk; *Bmp2/4*, *Smad6/7*, *Nkx-A* and *Nk4* in the ventral trunk; Figs 1–3 and S2–S4) and a concomitant anterior extension of posterior identity (*Cdx*, *Zf115*, *Msxb*, *Klf1/2/4* and *Nkx-C*; Figs 1–3 and S2–S5), suggesting that the trunk epidermis was respecified as tail epidermis. In the CNS, the situation is less extreme: posteriorization was observed in the anteriormost region (ectopic expression anteriorly for *Six1/2* and *Six3/6*; Figs 2 and 3 and S3 and S4) and within the tail nerve cord (loss of *Hox1* in the anterior tail nerve cord and anterior ectopic expression of *Hox12*; Figs 2 and S3 and S4). Inhibition of cWnt led to downregulation of *Msxb*, *Klf1/2/4* and *Nkx-C* but not of *Achaete-scute a-like2* in the tail neurogenic midlines (Figs 7 and S7), and only to downregulation of *Hox12* in the epidermis (Fig 5). In the CNS, the anteriormost and posteriormost regions were affected as revealed by the repression of *Six1/2*, *Six3/6* and *Hox12* (Fig 5). The above patterning defects triggered by cWnt are also possibly at play in the other germ layers, endoderm and mesoderm (Figs 2 and S3 and S4), as observed in some vertebrates [53,54].

### Traces of ancestral cWnt patterning function are visible in ascidian embryos

Data from cWnt activation together with the expression of *Wnt5* and *Wnt6* posteriorly and of Wnt antagonists (*Sfrp1/5*, *Ror-a* and *Ror-b*) anteriorly fit with a global view of graded Wnt activity from posterior to anterior. These data are in agreement with the proposed ancient role for cWnt signaling in patterning the AP axis during early embryonic development, at least at the base of the deuterostomes [22]. In particular, the ascidian epidermis that contains neurogenic domains (forming ESNs) is highly regionalized along the AP axis. While we are not aware of similar organization in vertebrates, with the exception of specific regions such as the amphibian cement gland, similarities might be drawn with the hemichordate neurogenic ectoderm whose AP pattern is regulated by Wnt signaling [22].

**Fig 9:**
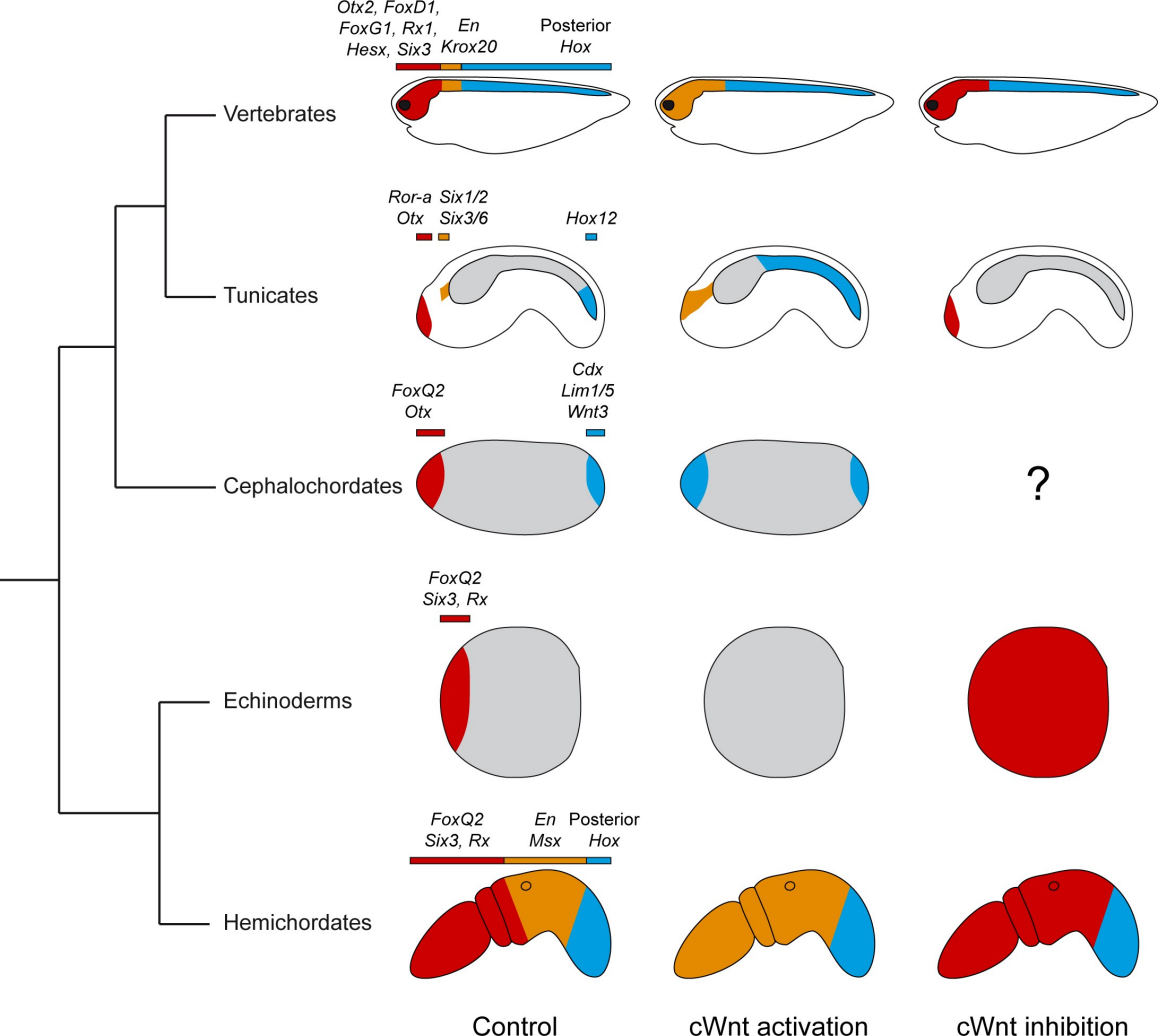
Summary of phenotypic effects of cWnt modulation on ectoderm AP patterning in deuterostomes. Schematic summary of data resulting from functional studies in vertebrates, amphioxus, echinoderms and hemichordate [10,18–22,55–59]. Note that the color code (red: anterior, orange: intermediate, blue: posterior) depicts regional domains along the AP axis and does not necessarily imply homology. Some of the key patterning genes whose expression has been determined in functional studies are listed above each domain.

Our results from cWnt activation are at first glance similar to what has been observed in other metazoans–repression of anterior identities and promotion of more posterior identities. We would like to discuss these observations by combining cWnt inhibition data and by restricting our comparisons to deuterostomes (Fig 9).

#### Anterior repression

The fact that anterior identity is incompatible with cWnt activity is well documented in deuterostomes (reviewed in [55]). Studies in different species from vertebrates, amphioxus, echinoderms and hemichordates have shown that cWnt activation abolishes anterior identity [18–22,56–59], as we observed for *Ciona*. While cWnt inhibition experiments have not been reported from amphioxus, data from vertebrates, echinoderms and hemichordates indicate that inhibiting cWnt is sufficient to enlarge the anterior of the embryo at the expense of more posterior territories (forebrain identity enlarged towards hindbrain in the vertebrate CNS). We did not detect such an effect when we examined anteriormost markers (*Islet* and *Ror-a* in Fig 5). This suggests that, in *Ciona*, cWnt inhibition is likely necessary but not sufficient to define the anterior identity. A tempting hypothesis involving inhibition of additional pathways will require further experimentation. However, analysis of *Etr* expression at neural plate stages (that labels the palp forming region; see Fig 6) was suggestive of an anteriorization upon cWnt inhibition (ectopic expression in lateral cells that are precursors of aATENs, ESNs located posterior to the palps at tailbud stages). This result needs to be confirmed with additional AP markers, a deeper scrutiny of the anterior ectoderm and lineage tracing experiments. If confirmed, this would indicate that patterning of the very anterior end of the *Ciona* embryo may be conserved with other deuterostomes.

#### Posteriorization

Posteriorization has been best studied in vertebrates and hemichordates where intermediate AP marker expression (hindbrain in vertebrates) is shifted anteriorly (in the fore-/mid-brain region in vertebrates) upon cWnt activation while more posterior markers (spinal cord in vertebrates) are not affected. The anterior extension of the *Six1/2* and *Six3/6* domains that we have observed in *Ciona* might be similar despite the fact that *Six3* marks the territory whose formation is inhibited by cWnt in other deuterostomes. We have not performed lineage tracing experiments but our results (ectopic expression of *Six1/2* and *Six3/6* at tailbud stages in Figs 3 and S4; loss of *Etr* at neural plate stages in Fig 6) are suggestive of ectopic *Six* gene expression in the epidermis immediately anterior to the sensory vesicle and in the palp region. Reciprocally, *Six1/2* and *Six3/6* expression is Wnt dependent and their loss upon cWnt inhibition might be associated with an anteriorization as discussed above. A conservation of graded cWnt activity to pattern the anterior would thus be found with vertebrates/hemichordates, but without a strict correspondence between the AP domains regulated by cWnt. Interestingly, a recent study has elegantly described specification mechanisms for these two sub-domains of the anterior neural plate border: the palp precursor region (medial at neural plate stages, then at the anterior tip at tailbud stages) and the aATENs precursor region (lateral at neural plate stages, then posterior to the palps at tailbud stages) [52]. It has been shown that two transcription factors control formation of these domains, *FoxC* (palps) and *Six1/2* (aATENs), and a reciprocal repression between them ensures proper fate segregation. cWnt could thus act before these interlocking regulatory interactions by specifying the aATENs domain.

By contrast, we do not consider that the posteriorization within the *Ciona* caudal nerve cord (ectopic *Hox12* expression) is comparable to the vertebrate/hemichordate case where the initial definition of the posteriormost part of the embryo is Wnt independent. The cWnt requirement for *Hox12* expression at the tip of the tail, both in the epidermis and in the nerve cord, where *Wnt5* is expressed [24,40] might, however, bear some similarity with the regulation of the posterior ectoderm by Wnt5 in sea urchin [60].

### What patterns the ascidian ectoderm AP axis?

The marked difference between activation (dramatic posteriorization phenotypes) and inhibition (discrete and limited phenotypes) of cWnt signaling was rather puzzling. An obvious explanation could be the incomplete inhibition of the pathway. TcfΔC has been previously used to inhibit endomesoderm formation in *Ciona* [25] and we have shown that it can suppress the action of the Gsk3 inhibitor 1-azakenpaullone (Fig 5); and Sfrp1/5 led to similar effects in the experiments presented here. The activation data that we have presented (Figs 1 and S2) show that the ectoderm is responsive to cWnt signaling for a prolonged period of time during gastrulation and neurulation, and cWnt signaling might consequently be ongoing during this time window (only around 4hrs in *Ciona* developing at 18°C [61]). We thus tested various combinations of the two ectodermal drivers (pFog and pFt), a strong ubiquitous driver (pEf1α [62,63]) as well as combinations of TcfΔC and Sfrp1/5 (S8 Fig). This did not lead to a dramatically stronger repression of the genes that we have tested. While we cannot rule out that cWnt is active in the ectoderm before our earliest driver (pFog: 16-cell stage), we conclude that a partial inhibition of cWnt is not the most likely explanation for the modest phenotypes that we have observed.

In addition to Wnt signaling, several pathways (Fgf, retinoic acid, Shh) participate in patterning the CNS of the vertebrate embryo [56,64–66]. In *Ciona*, while retinoic acid regulates AP identity of both the CNS and the epidermis at the level of the anterior tail, Fgf regulates tail tip identity of the epidermis but also CNS patterning at various places (tail tip, posterior sensory vesicle, pigment cells, anterior neural plate border) [24,40,67–70]. Fgf signaling is thus likely to interact with cWnt and may act as a redundant signal that compensates for the loss of Wnt signaling in our experiments; we aim at testing their respective functions in future experiments.

A major outcome of cWnt signaling is the regulation of gene expression and transcriptional reporters containing Tcf binding sites have been used as proxies to determine cWnt activity. We have used a reporter previously described in *Ciona* [40] and found the same global conclusions: reporter activity could be detected in endomesoderm derivatives and in the neurogenic tail ventral midline (S1 Table). We also detected activity in the posterior dorsal midline. In addition, our quantification of reporter activity showed that while endomesodermal activity was detected in a large majority of the embryos, epidermal activity was found, at best, in around 10% of the embryos. Furthermore, this reporter was not active in the CNS regions where we functionally uncovered a role for Wnt signaling. This suggests that cWnt activity in the ectoderm may be very low or possibly at levels undetectable by the reporter used, or that this reporter may not be a faithful readout of cWnt in the ectoderm.

Finally, a major explanation for the modest roles of cWnt in ectoderm AP patterning is likely to stem from the mosaic development of ascidians. In particular, it is well known that the binary AP difference in the ectoderm occurs as early as the 8-cell stage between the trunk and the tail ectoderm precursors, and that FoxA-a acts as an anterior determinant [26,28,71,72]. cWnt might thus be involved, possibly together or redundantly with other signals, in refining this basic pattern. For example, both *Wnt5* and *Wnt6* are expressed posteriorly and could participate in the definition of the posteriormost CNS and caudal PNS. *Wnt6* is also expressed transiently in the anterior neural plate border similarly to *Six3/6* and could play a role in the patterning of this region of the embryo [36,37]. Further combinatorial and targeted experiments will be required to definitively determine the precise function of Wnt signaling in ectoderm patterning.

### cWnt as a new actor in caudal PNS formation

While cWnt is not essential for caudal PNS formation, we have uncovered two distinct functions. First, cWnt appears to interact with Bmp signaling to position within the embryo the ventral neurogenic midline, by regulating the expression of the gene *Msxb* (S6 Fig). This is not the only mechanism involved since the expression of *Achaete-scute a-like2*, another early midline gene, is Wnt independent. It would be interesting to uncover and compare the mechanisms that initiate the transcription of both genes in the tail ventral ectoderm through the study of their *cis*-regulatory DNAs. The timed activation of cWnt allowed us to uncover a later function for cWnt that is independent of the posteriorization; cWnt repressed ESN formation (Fig 1). It is well known that Notch signaling regulates the number of ESNs that form in the caudal midlines and launches a proneural transcriptional cascade [43,73–75]. It will thus be important to determine whether cWnt interacts with this GRN and at which level.

## Methods

### Embryo obtention and manipulation

Ripe adults of *Ciona intestinalis* (formerly referred to *Ciona intestinalis* type B [32]) were provided by the Centre de Ressources Biologiques Marines in Roscoff (EMBRC-France). Embryo obtention and electroporation were performed as described [72]: 50 μg of each plasmid DNA were used in a 350 μl electroporation volume placed in a 4 mm cuvette and a single pulse of 25V for 32 ms was applied using an ECM830 electroporator (BTX, Harvard Bioscience). Stock solutions of 1-azakenpaullone (191500, Calbiochem, Merck) and BIO (361550, Calbiochem, Merck) were prepared at 10 mM in DMSO. Dilutions were made in sea water just before use at the concentration indicated in the text. Embryo staging and neural plate description were performed according to [50,61].

### Molecular constructs

We have used several previously reported constructs: pFog>Noggin, pFog>Admp and pFog>Venus [43], pFt>ΔN-β-catenin and p12xTcf>nlsLacZ [40]. The other constructs were generated using dedicated Gateway vectors [76]. The activity of the following promoters has been previously described: pFog (pan-ectodermal from the 16-cell stage) [49], pFt (pan-ectodermal from the 64-cell stage) [40] and pEf1α (ubiquitous from early gastrula stages) [62,63]. While the first two were available in Gateway vectors, the last one was introduced following PCR amplification (Forward primer: AAAAAGCAGGCTTTGCTTTACCATCGCGTGACG, reverse primer: AGAAAGCTGGGTTTTGGAAGGTTGGGGTTAACC) using pSPCiEF1α>Cas9 [77] as a template. We have used entry clones containing the coding sequence of ΔN-β-catenin (generated by a BP reaction from pFt>ΔN-β-catenin [40]), TcfΔC (generated by PCR from pRN3-TcfΔC [25]. Forward primer: AAAAAGCAGGCTCAGAAAAAATGCCTCAGTTAAACTCGGA, reverse primer: AGAAAGCTGGGTTCATGGCCGACTTGGTTTG), Sfrp1/5 (generated by RT-PCR from initial tailbud stages *C*. *robusta* RNA. Forward primer: CAGAAAAAATGGGATCGTGGATAAAAGGA, reverse primer: TTATCTCCCAGCAGAACCAGTG) and Wnt5 [78] (clone cien109569).

### *In situ* hybridization, alkaline phosphatase, and X-gal staining

Whole mount *in situ* hybridization and X-gal staining (detection of β-galactosidase activity following p12xTcf>nlsLacZ electroporation) were performed as previously described [26,79]. Dig-labeled probes were synthesized from *C*. *robusta* clones described in previous publications [26,80,81], obtained from cDNA libraries [78,82] or generated by cloning RT-PCR products (from initial tailbud stages embryonic RNA) into pGEM-T Easy (Promega) (S2 Table). Effects on gene expression were analyzed for each marker on 15–40 embryos for inhibitor treatments and 40–70 electroporated embryos (the number of independent experiments is indicated in the figure legend). Embryos treated with DMSO or electroporated with pFog>Venus were used as controls.

Colorimetric detection of endogenous alkaline phosphatase activity was adapted from [13]: embryos were fixed 10 min at room temperature in sea water containing 5% formaldehyde, washed twice 10 min in TMNTw (100 mM NaCl, 50 mM MgCl_2_, 100 mM Tris pH 9.5, 0.1% Tween20) and stained in TMNTw containing 3.3 μl/ml of NBT (50 mg/ml) and 1.75 μl/ml of BCIP (50 mg/ml).

All pictures were taken from embryos in PBTw using a Zeiss Discovery V20 dissecting scope equipped with an AxioCam ERc5s digital camera. Image panels and figures were constructed with Adobe Photoshop and Adobe Illustrator.

### Gene model identifiers

The genes described in this study are represented by the following gene models in the KH2012 *C*. *robusta* assembly: genes whose expression has been analyzed by *in situ* hybridization (see S2 Table), *Fog* (KH.C10.574), *Ft* (KH.C11.299), *Ef1α* (KH.C14.52), *Noggin* (KH.C12.562), *Admp* (KH.C2.421), *β-catenin* (KH.C9.53) *Tcf* (KH.C6.71), *Sfrp1/5* (KH.L171.5), and *Wnt5* (KH.L152.45).

## Supporting information

S1 Fig Ectopic endoderm formation following cWnt activation. Embryos were treated with 10 μM 1-azakenpaullone (AZA) at the stage indicated on the picture and left to develop until late tailbud stages (stages 24/25) when endogenous alkaline phosphatase staining was performed to reveal endoderm formation. Compared to control DMSO-treated embryos (A), 1-azakenpaullone-treated embryos at the 8-cell (B) and the 16-cell (C) stages presented ectopic staining; unstained cells are presumably muscle cells. Later treatments (D-F) did not lead to ectopic endoderm formation although the shape of the embryos was strongly affected. Embryos are oriented with dorsal to the top and anterior to the left. Experiment performed once.
(PDF)

S2 Fig Timing of ectoderm sensitivity to cWnt activation. Embryos were treated with DMSO (A), 10 μM 1-azakenpaullone (B) or 2.5 μM BIO (C) from initial tailbud stages (stage 17) until fixation at late tailbud stages (stage 24). Expression of *Zf115* was determined by *in situ* hybridization and was found unchanged by activating cWnt. (D-S) Embryos were treated with 10 μM 1-azakenpaullone for 30 minutes at the stage indicated on the left of the figure before being extensively washed in seawater. They were then fixed at late tailbud stages (stage 24) and the expression of *Etr* (D-K) and *Zf115* (L-S) was analyzed by *in situ* hybridization. Robust ectopic *Etr* expression in the ventral trunk (white arrowheads) was observed for treatments at stages 10 and 11. Far fewer ectopic *Etr* positive cells were observed for treatments at stages 12 and 13, and no ectopic staining was observed for later treatments. Anterior palp neuron staining (black arrows) was abolished for all treatments except the last one for which a strong downregulation was observed (H). *Zf115* was ectopically expressed in trunk epidermis for all treatments but its anterior limit (red arrow) was shifted posteriorly as the treatment was delayed. The *Zf115* negative region in M corresponds to the open neural tube. Embryos are oriented with dorsal to the top and anterior to the left. Experiment performed once.
(PDF)

S3 Fig Effects of activating cWnt using small molecule inhibitors on a collection of markers at early tailbud stages. Embryos were treated with 10 μM 1-azakenpaullone or 2.5 μM BIO from stage 10 (initial gastrula), fixed at stage 19/20 (early tailbud) and processed for *in situ* hybridization to determine the expression pattern of the genes indicated on the left of each panel. The embryos presented in Fig 2 are included in this figure. Embryos are oriented with dorsal to the top and anterior to the left. Number of experiments: one for *Smad6/7*, *Nkx-A*, *Islet*, *Ror-a*, *Trp*, *Mytf*, *Gbe1*, *Hox1*, *KH*.*C7*.*391*, *Hox12*, *FoxF*, *Zf115*, *Bra* and *Ferritin*; two for *Klf1/2/4*, *Msxb*, *Nk4*, *Bmp2/4*, *Otx* and *Six3/6*.
(PDF)

S4 Fig Effects of increasing concentrations of 1-azakenpaullone. Embryos were treated with various concentrations (indicated on the figure) of 1-azakenpaullone (AZA) from stage 10 (initial gastrula) to early tailbud stages (stages 20/21) for *in situ* hybridization or swimming larval stages for alkaline phosphatase histochemistry. Embryos are oriented with dorsal to the top and anterior to the left. Experiment performed once.
(PDF)

S5 Fig Overexpression of Wnt5 or ΔN-β-catenin promotes ectopic posterior marker expression in the trunk. Embryos were electroporated with either pFog>Wnt5 or pFt>ΔN-β-catenin and fixed at early tailbud stages (stages 18/19) to analyze *Msxb*, *Klf1/2/4* and *Nkx-C* expression by *in situ* hybridization. In all cases, ectopic expression was detected in the trunk ventral midline (white arrowheads). Experiment performed once for pFog>Wnt5 and twice or more for pFt>ΔN-β-catenin for each probe.
(PDF)

S6 Fig Regulation of *Msxb* expression by the combined action of Wnt and Bmp signals. (A) Control unelectroporated embryo. (B-F) Embryos were electroporated with pFog>Noggin (B), pFog>Admp (C), pFt>ΔN-β-catenin (D), pFog>Noggin + pFt>ΔN-β-catenin (E) and pFog>Admp + pFt>ΔN-β-catenin (F), and fixed for *in situ* hybridization at initial tailbud stages (stage 18) for *Msxb*. Ectopic staining is highlighted with the white arrowheads. Experiment performed once.
(PDF)

S7 Fig Tail PNS genes require active Wnt signaling. Embryos were electroporated with pFog>Venus (A, D), pFt>Sfrp1/5 (B, E) and pFog>TcfΔC (C, F), and fixed for *in situ* hybridization at initial tailbud stages (stage 18) for *Nkx-C* (A-C) and at early mid tailbud stages (stage 21) for *Klf1/2/4* (D-F). Expression of both genes is downregulated when Wnt signaling is inhibited. Embryos are oriented with dorsal to the top and anterior to the left. Experiment performed four times or more for both probes.
(PDF)

S8 Fig Effects of plasmid co-electroporation on gene expression. Each graph represents a single electroporation experiment analyzed by *in situ* hybridization for the gene and at the stage indicated at the top. Embryos were scored for change in gene expression pattern as "normal" (blue) or "repression" (grey).
(PDF)

S1 Table cWnt reporter activity. Embryos were electroporated with the p12xTcf>nlsLacZ reporter construct [40] and fixed at the indicated stage for X-gal staining. Embryos were scored according to the location of the staining.
(PDF)

S2 Table List of cDNA clones used for *in situ* hybridization. (PDF)
