# Supplementary figures and images for: Antero-posterior ectoderm patterning by canonical Wnt signaling during ascidian development
Source: PLoS Genet. 2019 Mar 29;15(3):e1008054. doi: 10.1371/journal.pgen.1008054 (PMC6457572; doi:10.1371/journal.pgen.1008054)

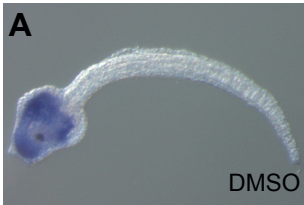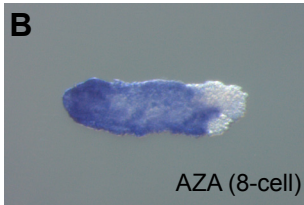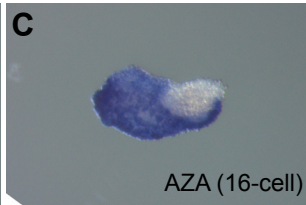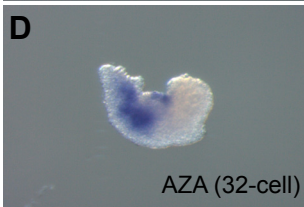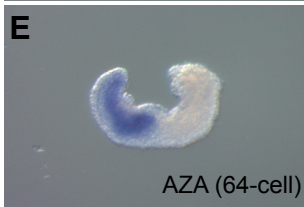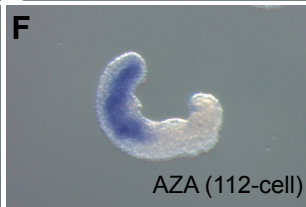

Supplement: S1 Fig — Embryos were treated with 10 μM 1-azakenpaullone (AZA) at the stage indicated on the picture and left to develop until late tailbud stages (stages 24/25) when endogenous alkaline phosphatase staining was performed to reveal endoderm formation. Compared to control DMSO-treated embryos (A), 1-azakenpaullone-treated embryos at the 8-cell (B) and the 16-cell (C) stages presented ectopic staining; unstained cells are presumably muscle cells. Later treatments (D-F) did not lead to ectopic endoderm formation although the shape of the embryos was strongly affected. Embryos are oriented with dorsal to the top and anterior to the left. Experiment performed once. (PDF) [file pgen.1008054.s001.pdf]

30 min pulse of 10  $\mu$ M 1-azakenpaulone

DMSO from stage 17      10  $\mu$ M 1-azakenpaulone from stage 17      2.5 $\mu$ M BIO from stage 17

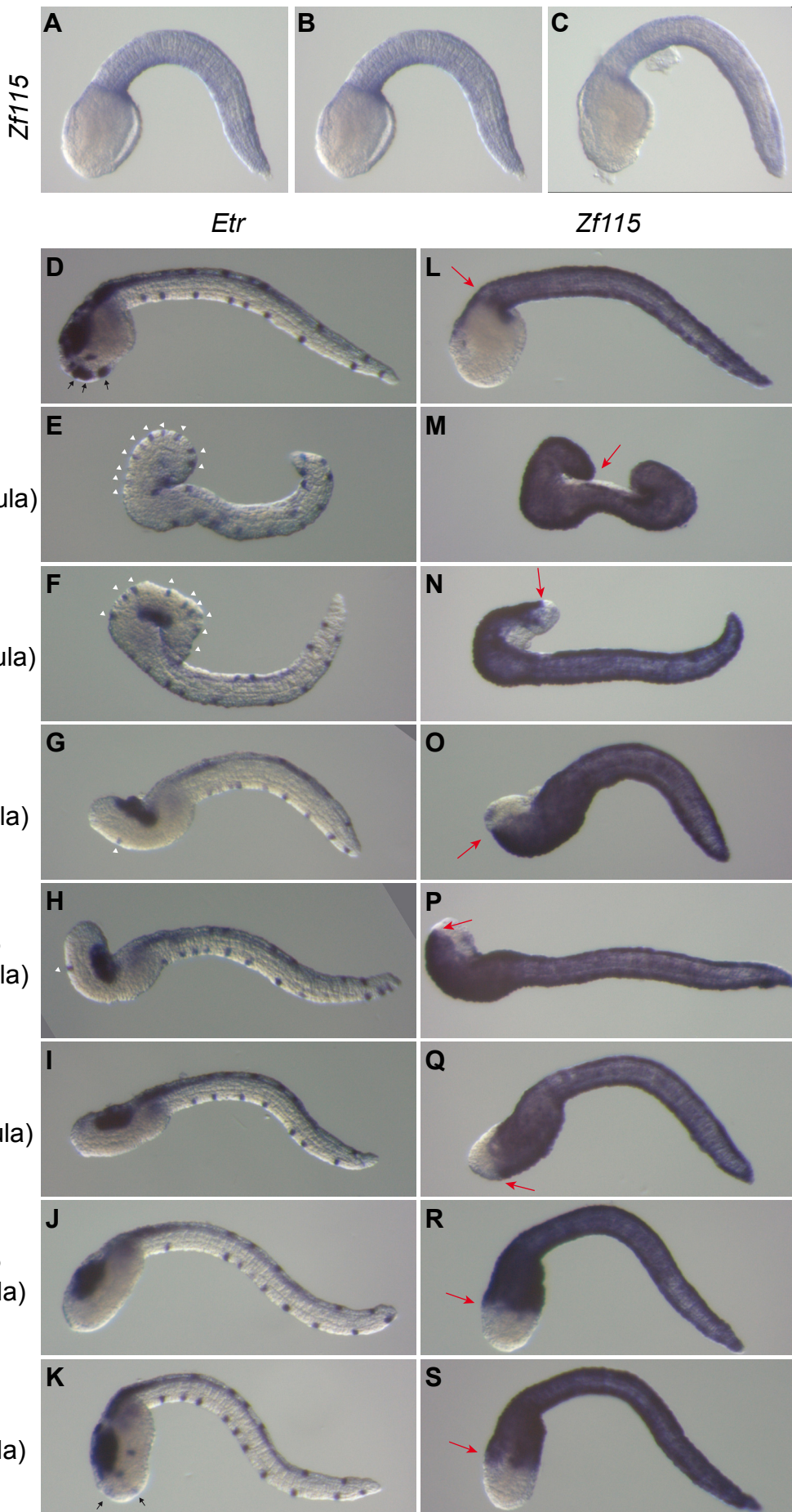

Supplement: S2 Fig — Embryos were treated with DMSO (A), 10 μM 1-azakenpaullone (B) or 2.5 μM BIO (C) from initial tailbud stages (stage 17) until fixation at late tailbud stages (stage 24). Expression of Zf115 was determined by in situ hybridization and was found unchanged by activating cWnt. (D-S) Embryos were treated with 10 μM 1-azakenpaullone for 30 minutes at the stage indicated on the left of the figure before being extensively washed in seawater. They were then fixed at late tailbud stages (stage 24) and the expression of Etr (D-K) and Zf115 (L-S) was analyzed by in situ hybridization. Robust ectopic Etr expression in the ventral trunk (white arrowheads) was observed for treatments at stages 10 and 11. Far fewer ectopic Etr positive cells were observed for treatments at stages 12 and 13, and no ectopic staining was observed for later treatments. Anterior palp neuron staining (black arrows) was abolished for all treatments except the last one for which a strong downregulation was observed (H). Zf115 was ectopically expressed in trunk epidermis for all treatments but its anterior limit (red arrow) was shifted posteriorly as the treatment was delayed. The Zf115 negative region in M corresponds to the open neural tube. Embryos are oriented with dorsal to the top and anterior to the left. Experiment performed once. (PDF) [file pgen.1008054.s002.pdf]

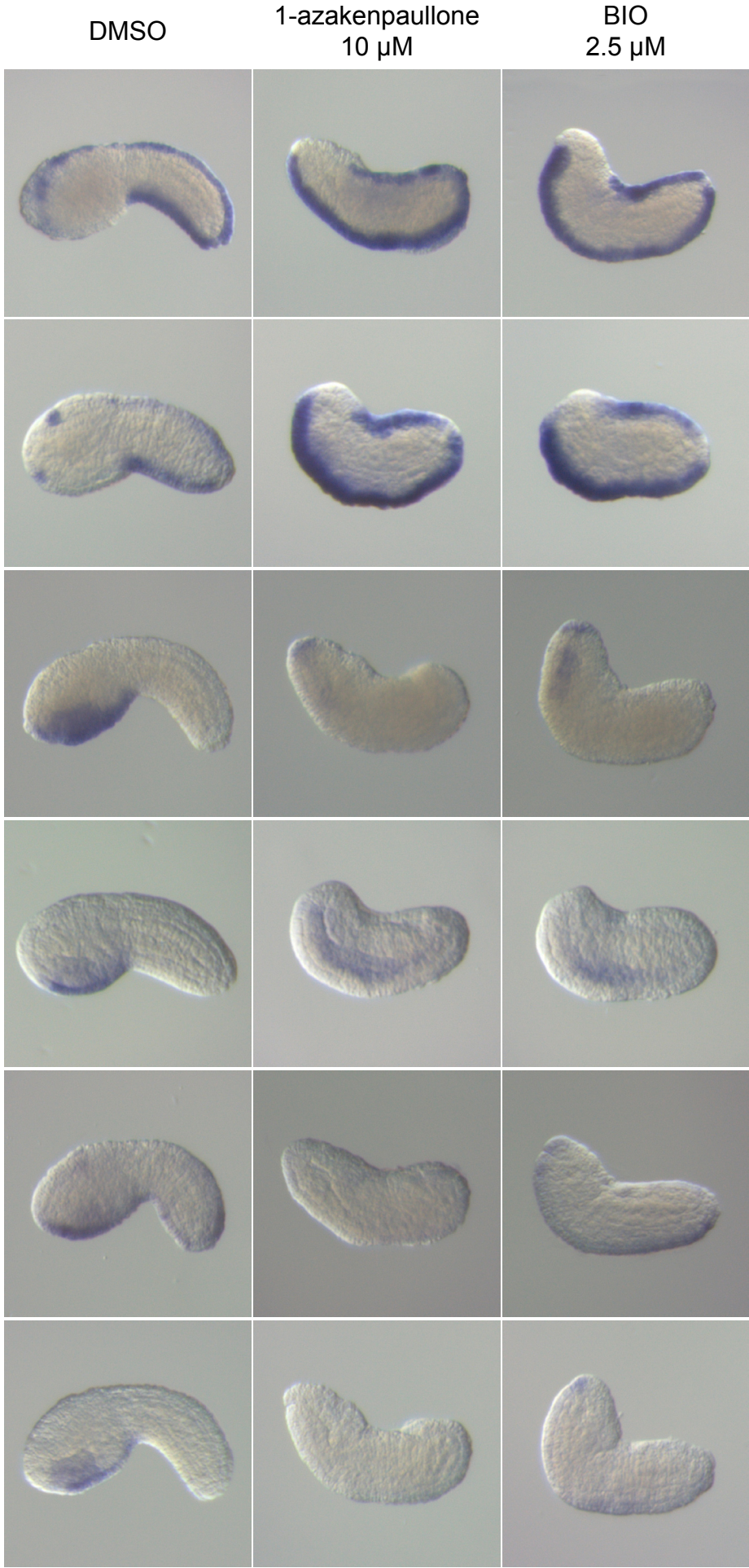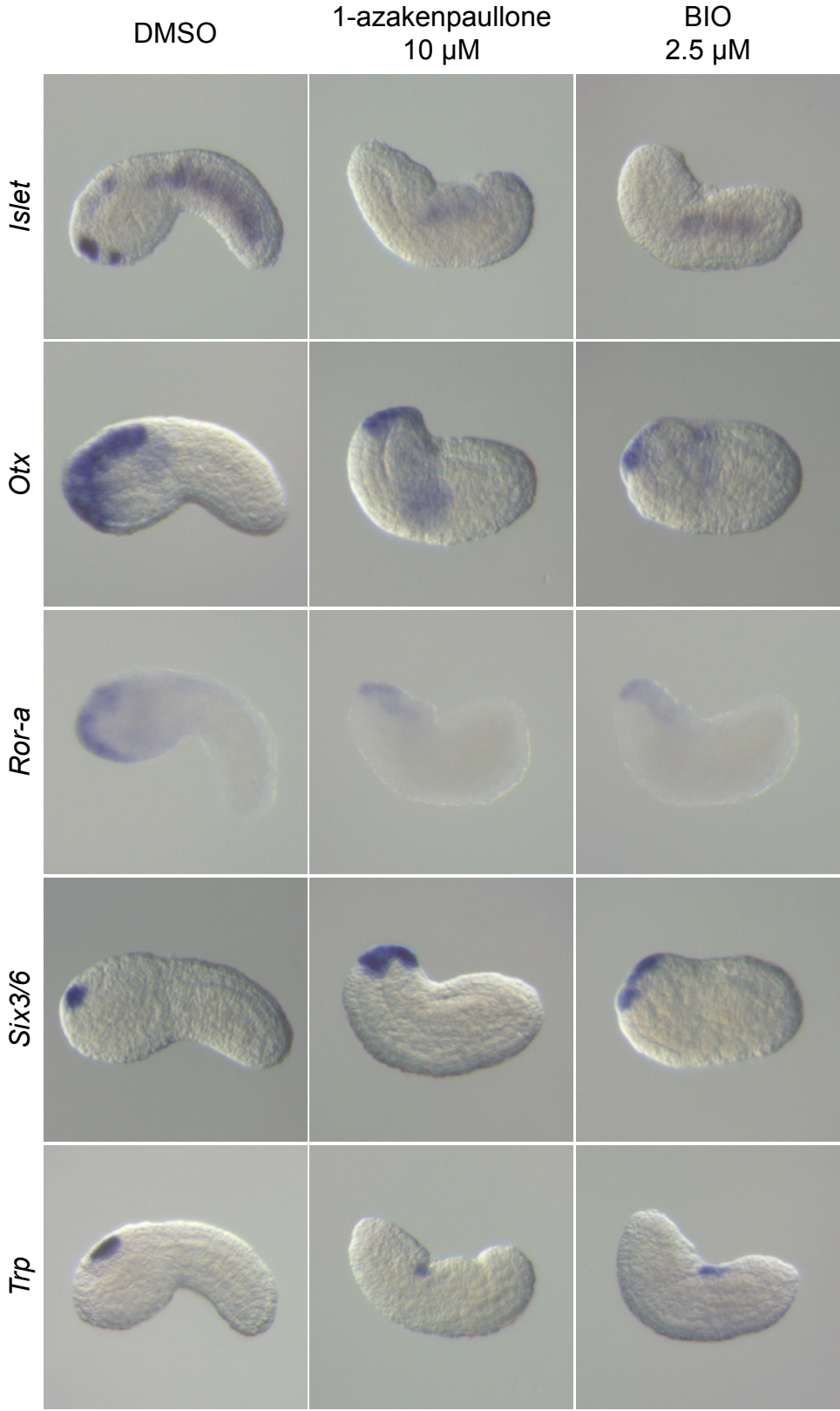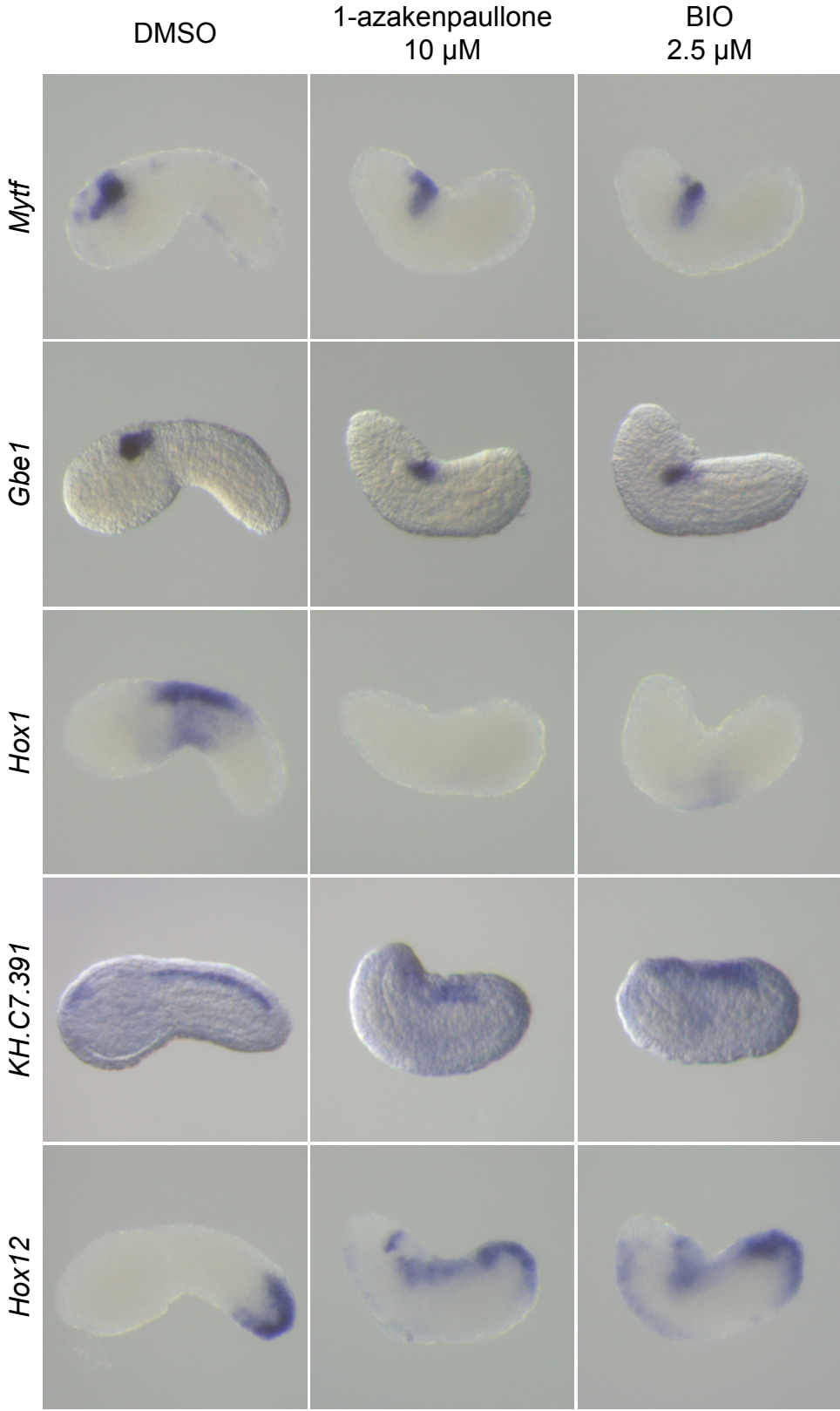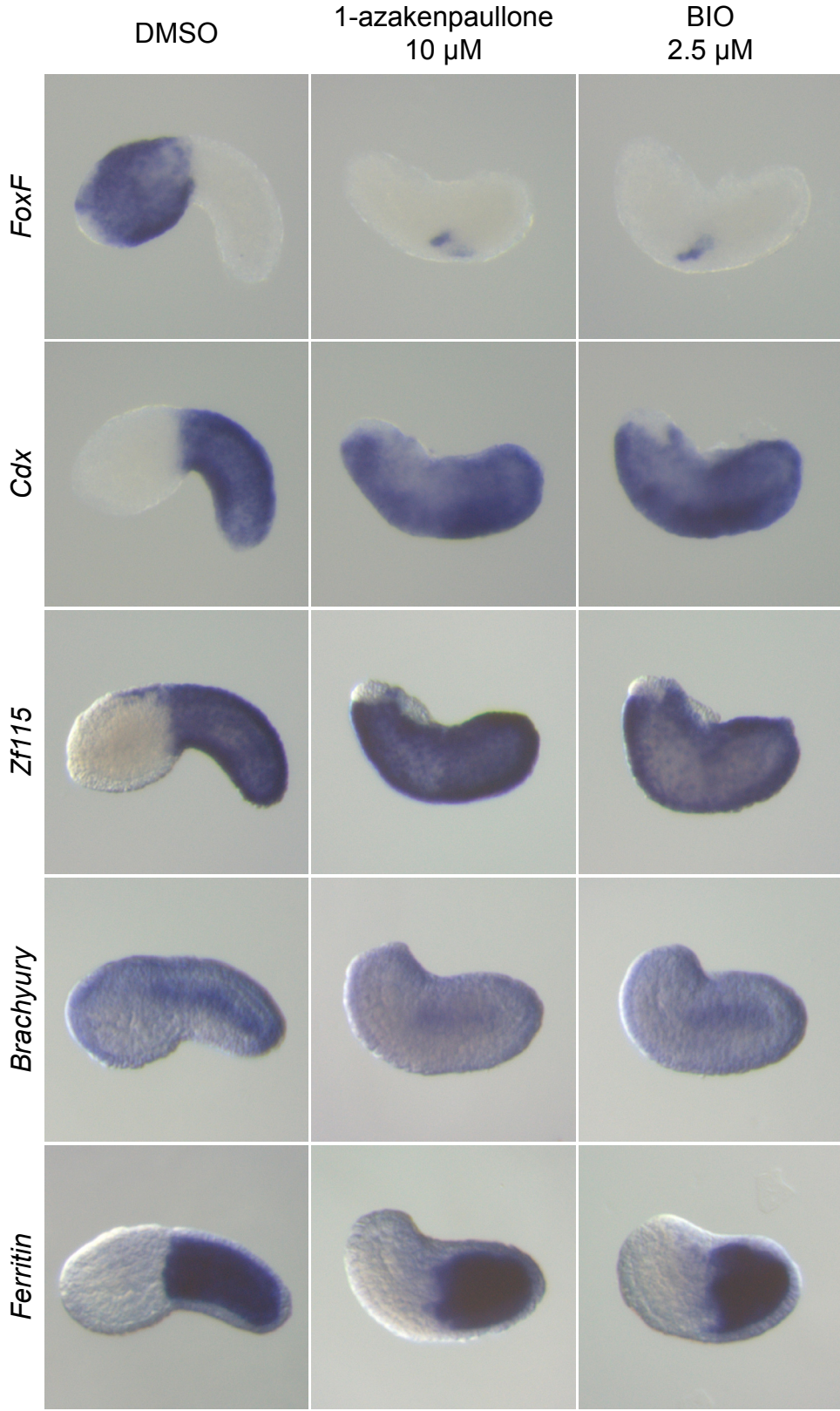

Supplement: S3 Fig — Embryos were treated with 10 μM 1-azakenpaullone or 2.5 μM BIO from stage 10 (initial gastrula), fixed at stage 19/20 (early tailbud) and processed for in situ hybridization to determine the expression pattern of the genes indicated on the left of each panel. The embryos presented in Fig 2 are included in this figure. Embryos are oriented with dorsal to the top and anterior to the left. Number of experiments: one for Smad6/7, Nkx-A, Islet, Ror-a, Trp, Mytf, Gbe1, Hox1, KH.C7.391, Hox12, FoxF, Zf115, Bra and Ferritin; two for Klf1/2/4, Msxb, Nk4, Bmp2/4, Otx and Six3/6. (PDF) [file pgen.1008054.s003.pdf]

Control

pFog >Wnt5

pFt > $\Delta$ N- $\beta$ -catenin

*Msxb*

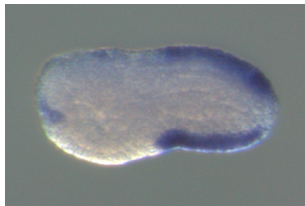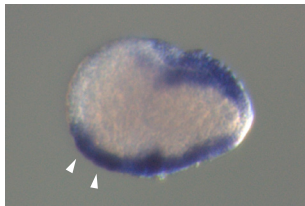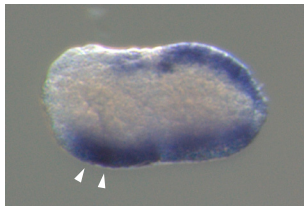

*Klf1/2/4*

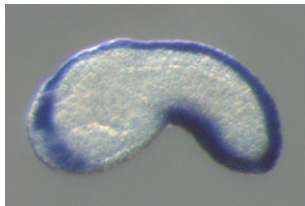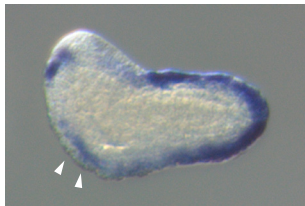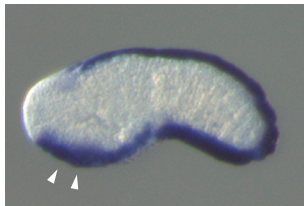

*Nkx-C*

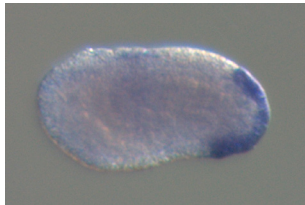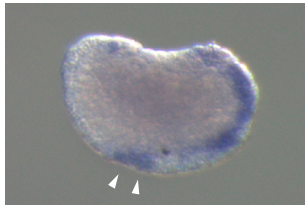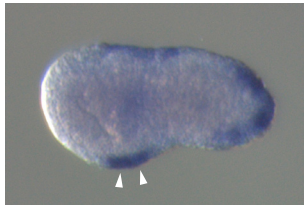

Supplement: S5 Fig — Embryos were electroporated with either pFog>Wnt5 or pFt>ΔN-β-catenin and fixed at early tailbud stages (stages 18/19) to analyze Msxb, Klf1/2/4 and Nkx-C expression by in situ hybridization. In all cases, ectopic expression was detected in the trunk ventral midline (white arrowheads). Experiment performed once for pFog>Wnt5 and twice or more for pFt>ΔN-β-catenin for each probe. (PDF) [file pgen.1008054.s005.pdf]

pFt >  
 $\Delta$ N- $\beta$ -catenin

Control

pFog >Noggin

pFog >Admp

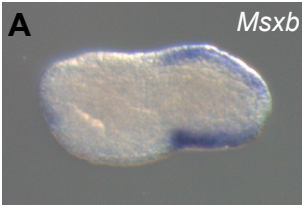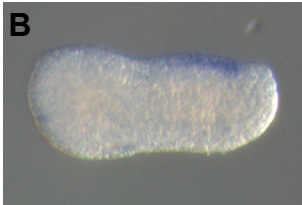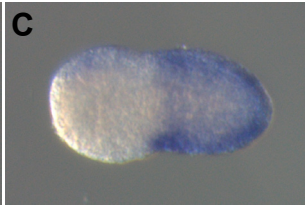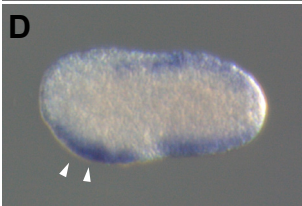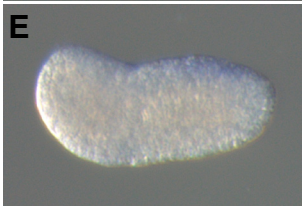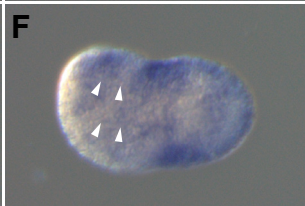

Supplement: S6 Fig — (A) Control unelectroporated embryo. (B-F) Embryos were electroporated with pFog>Noggin (B), pFog>Admp (C), pFt>ΔN-β-catenin (D), pFog>Noggin + pFt>ΔN-β-catenin (E) and pFog>Admp + pFt>ΔN-β-catenin (F), and fixed for in situ hybridization at initial tailbud stages (stage 18) for Msxb. Ectopic staining is highlighted with the white arrowheads. Experiment performed once. (PDF) [file pgen.1008054.s006.pdf]

pFog >Venus

pFt >Sfrp1/5

pFog >Tcf $\Delta$ C

**A**

**B**

**C**

*Nkx-C*

**D**

**E**

**F**

*Klf1/2/4*

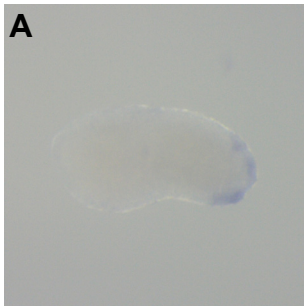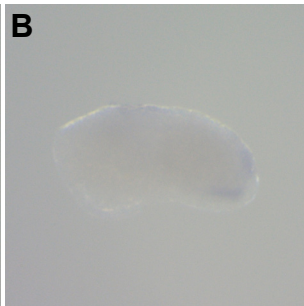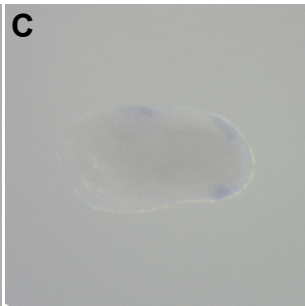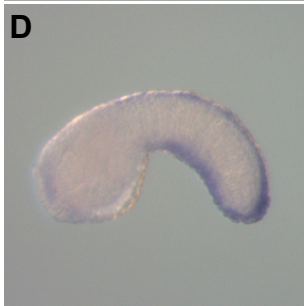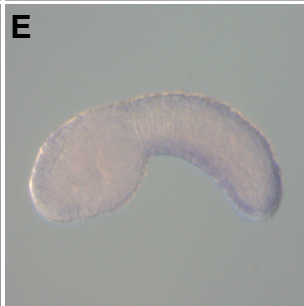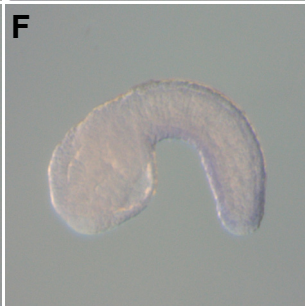

Supplement: S7 Fig — Embryos were electroporated with pFog>Venus (A, D), pFt>Sfrp1/5 (B, E) and pFog>TcfΔC (C, F), and fixed for in situ hybridization at initial tailbud stages (stage 18) for Nkx-C (A-C) and at early mid tailbud stages (stage 21) for Klf1/2/4 (D-F). Expression of both genes is downregulated when Wnt signaling is inhibited. Embryos are oriented with dorsal to the top and anterior to the left. Experiment performed four times or more for both probes. (PDF) [file pgen.1008054.s007.pdf]

*Msx*b (stage 18)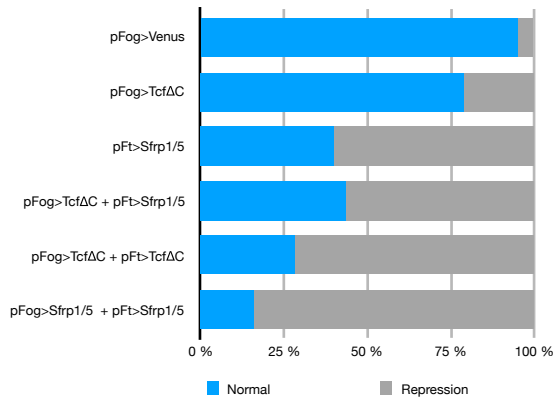*Msx*b (stage 18)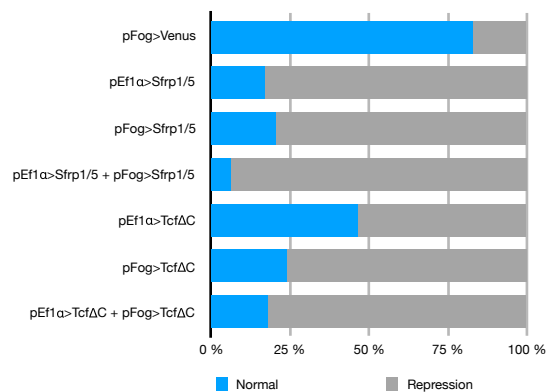*Klf1/2/4* (stage 21)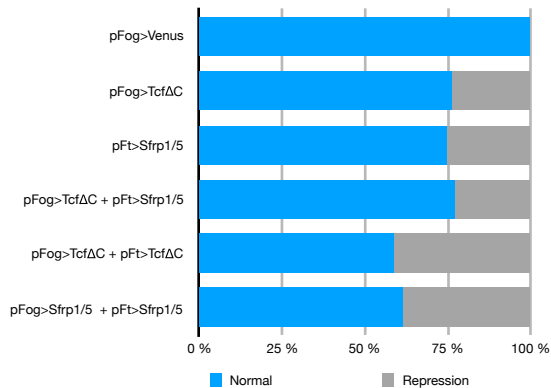*Klf1/2/4* (stage 21)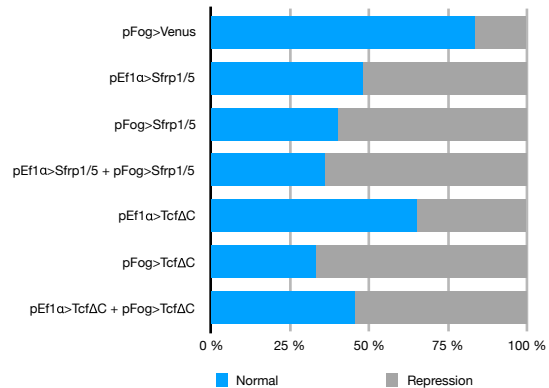*Six3/6* (stage 21)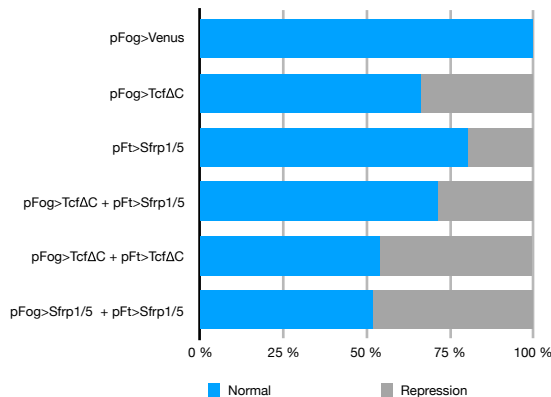*Cdx* (stage 21)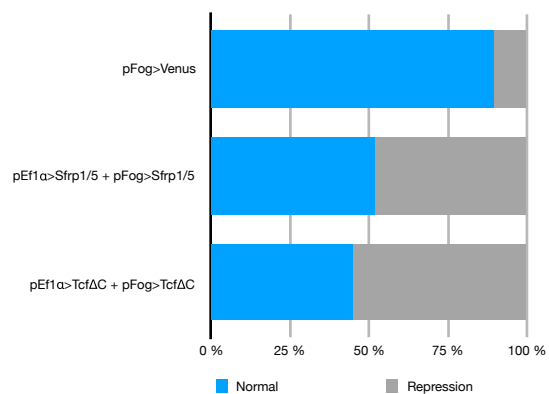

Supplement: S8 Fig — Each graph represents a single electroporation experiment analyzed by in situ hybridization for the gene and at the stage indicated at the top. Embryos were scored for change in gene expression pattern as "normal" (blue) or "repression" (grey). (PDF) [file pgen.1008054.s008.pdf]
