# Supplementary material for: Antero-posterior ectoderm patterning by canonical Wnt signaling during ascidian development
Source: PLoS Genet. 2019 Mar 29;15(3):e1008054. doi: 10.1371/journal.pgen.1008054 (PMC6457572; doi:10.1371/journal.pgen.1008054)
Supplement: S4 Fig — Embryos were treated with various concentrations (indicated on the figure) of 1-azakenpaullone (AZA) from stage 10 (initial gastrula) to early tailbud stages (stages 20/21) for in situ hybridization or swimming larval stages for alkaline phosphatase histochemistry. Embryos are oriented with dorsal to the top and anterior to the left. Experiment performed once. (PDF) [file pgen.1008054.s004.pdf]

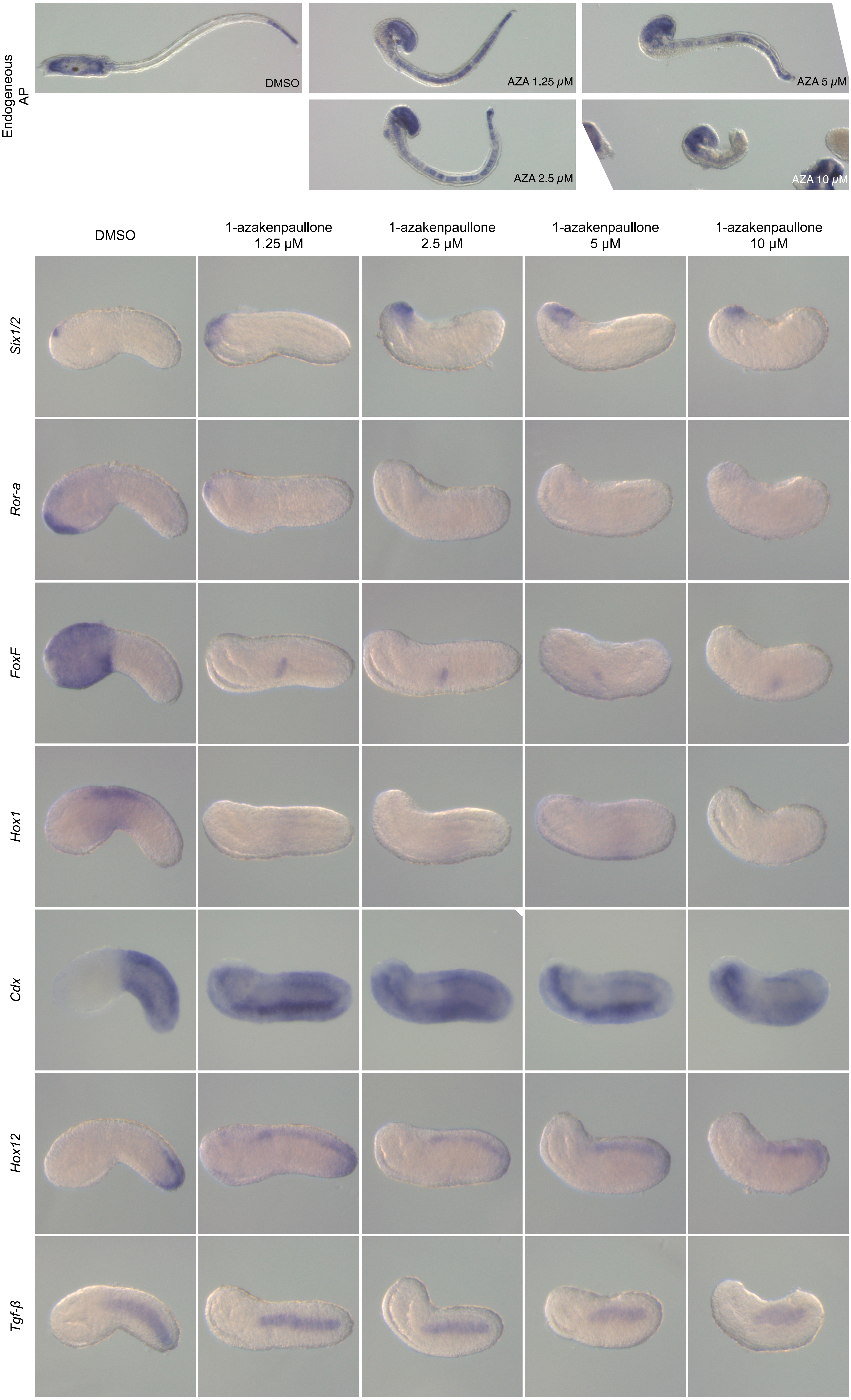
Endogeneous AP
DMSO
AZA 1.25 μM
AZA 5 μM
AZA 2.5 μM
AZA 10 μM
DMSO
1-azakenpaullone 1.25 μM
1-azakenpaullone 2.5 μM
1-azakenpaullone 5 μM
1-azakenpaullone 10 μM
Six1/2
Ror-a
FoxF
Hox1
Cdx
Hox12
Tgf-β
