# Supplementary material for: Antero-posterior ectoderm patterning by canonical Wnt signaling during ascidian development
Source: PLoS Genet. 2019 Mar 29;15(3):e1008054. doi: 10.1371/journal.pgen.1008054 (PMC6457572; doi:10.1371/journal.pgen.1008054)
Supplement: S1 Table — Embryos were electroporated with the p12xTcf>nlsLacZ reporter construct [40] and fixed at the indicated stage for X-gal staining. Embryos were scored according to the location of the staining. (PDF) [file pgen.1008054.s009.pdf]

cWnt reporter (p12xTcf>nlsLacZ)

			X-gal activity detected in		
**Stage of analysis**	**Number of experiments compiled**	**Number of embryos examined**	**endomesoderm**	**posterior ventral tail epidermis midline**	**posterior dorsal tail epidermis midline**
**Early gastrula (St. 11)**	1	198	38 %	0 %	0 %
**Gastrula/neurula (St. 12 to 16)**	3	572	64 %	7 %	0 %
**Early tailbud (St. 18 to 22)**	5	837	82 %	11 %	3 %
**Mid-tailbud (St. 23 to 24)**	3	528	67 %	4 %	3 %
**Late tailbud (St. 25 to 26)**	2	697	49 %	1 %	0 %
