# Supplementary material for: Antero-posterior ectoderm patterning by canonical Wnt signaling during ascidian development
Source: PLoS Genet. 2019 Mar 29;15(3):e1008054. doi: 10.1371/journal.pgen.1008054 (PMC6457572; doi:10.1371/journal.pgen.1008054)
Supplement: S2 Table — (PDF) [file pgen.1008054.s010.pdf]

In situ clones

Gene name	Gene ID (KH2012)	cDNA clone origin	Forward primer	Reverse primer
Msxb	KH.C2.957	cign067l18		
Nkx-C	KH.C1.922	citb089b03		
Klf1/2/4	KH.C5.154	citb012d14		
Achaete-scute a-like2	KH.L9.13	cien82323		
Etr	KH.C6.128	citb028e11		
Ap2-like2	KH.C7.43	cien223529		
Ror-a	KH.C8.101	cilv008g20		
KH.C7.391	KH.C7.391	cilv038e26		
FoxF	KH.C3.170	cicl007c02		
Hox12	KH.C7.472	cilv047h06		
Cdx	KH.C14.408	citb004h19		
Bmp2/4	KH.C4.125	cicl060n01		
Nk4	KH.C8.482	citb005h13		
Nkx-A	KH.C12.577	cicl048p22		
Zf115	KH.C1.927	cilv037e04		
Six3/6	KH.C10.367	cicl021e08		
Gbe1	KH.C6.224	08ZC09		
Trp	KH.C8.537	10ZF07		
Ferritin	KH.C2.36	05ZC04		
Tgf-β	KH.C3.724	cibd026d22		
Smad6/7	KH.C3.230	ciad037n07		
Otx	KH.C4.84	Hudson and Lemaire, 2001		
Islet	KH.L152.2	Giuliano et al., 1998		
Brachyury	KH.S1404.1	Corbo et al., 1997		
Mytf	KH.C1.274	RT-PCR	TGCTTGAAAACGAGATAAAAGCGA	ACACGCAGTAACTAAGCCCC
Hox1	KH.L171.16	RT-PCR	TAGCAGCAACACACGAAGGAT	TGTGTGACGTTACTCGCTGT
Six1/2	KH.C3.553	RT-PCR	TACAATCACGGCCTCAGAGC	TACTACACGTCACCGAAGCG
